# Systematic Review and Meta-Analysis of Seasonal Malaria Chemoprevention

**DOI:** 10.4269/ajtmh.23-0481

**Published:** 2023-12-11

**Authors:** Julie Thwing, John Williamson, Irene Cavros, Julie R. Gutman

**Affiliations:** ^1^ Malaria Branch, Center for Global Health, U.S. Centers for Disease Control and Prevention, Atlanta, Georgia;; ^2^ U.S. President’s Malaria Initiative, Malaria Branch, U.S. Centers for Disease Control and Prevention, Atlanta, Georgia

## Abstract

Seasonal malaria chemoprevention (SMC) for children under 5 years of age for up to four monthly cycles during malaria transmission season was recommended by the WHO in 2012 and has been implemented in 13 countries in the Sahel, reaching more than 30 million children annually. Malaria control programs implementing SMC have asked the WHO to consider expanding the age range or number of monthly cycles. We conducted a systematic review and meta-analysis of SMC among children up to 15 years of age and up to six monthly cycles. Twelve randomized studies were included, with outcomes stratified by age (< 5/≥ 5 years), by three or four versus five or six cycles, and by drug where possible. Drug regimens included sulfadoxine–pyrimethamine + amodiaquine, amodiaquine–artesunate, and sulfadoxine–pyrimethamine + artesunate. Included studies were all conducted in Sahelian countries in which high-grade resistance to sulfadoxine–pyrimethamine was rare and in zones with parasite prevalence ranging from 1% to 79%. Seasonal malaria chemoprevention resulted in substantial reductions in uncomplicated malaria incidence measured during that transmission season (rate ratio: 0.27, 95% CI: 0.25–0.29 among children < 5 years; rate ratio: 0.27, 95% CI: 0.25–0.30 among children ≥ 5 years) and in the prevalence of malaria parasitemia measured within 4–6 weeks from the final SMC cycle (risk ratio: 0.38, 95% CI: 0.34–0.43 among children < 5 years; risk ratio: 0.23, 95% CI: 0.11–0.48 among children ≥ 5 years). In high-transmission zones, SMC resulted in a moderately reduced risk of any anemia (risk ratio: 0.77, 95% CI: 0.72–0.83 among children < 5 years; risk ratio: 0.70, 95% CI: 0.52–0.95 among children ≥ 5 years [one study]). Children < 10 years of age had a moderate reduction in severe malaria (risk ratio: 0.53, 95% CI: 0.37–0.76) but no evidence of a mortality reduction. The evidence suggests that in areas in which sulfadoxine–pyrimethamine and amodiaquine remained efficacious, SMC effectively reduced malaria disease burden among children both < 5 and ≥ 5 years old and that the number of cycles should be commensurate with the length of the transmission season, up to six cycles.

## INTRODUCTION

Malaria caused by *Plasmodium falciparum* remains a major cause of ill health and death in sub-Saharan Africa.^1^ In the Sahel subregion of Africa, most childhood malaria morbidity and mortality occur during the rainy season, which lasts 3–4 months.^2^^,^^3^

In March 2012, the WHO recommended seasonal malaria chemoprevention (SMC) for children aged 3–59 months living in areas of highly seasonal malaria transmission (the majority of cases within a 4-month period) in the Sahel subregion of Africa.^4^ Seasonal malaria chemoprevention is defined as the monthly administration of up to four full treatment courses of an antimalarial medicine, usually sulfadoxine–pyrimethamine + amodiaquine (SP+AQ), to children in areas of highly seasonal transmission where the annual clinical attack rate was at least 0.1 episode per child and where SP and AQ were efficacious, beginning at the start of the transmission season.

The objective of SMC is to prevent malarial illness by maintaining therapeutic antimalarial drug concentrations in the blood throughout the period of peak seasonal malaria transmission. A systematic review and meta-analysis of SMC showed that it offers young children a high level of protection against clinical malaria and all-cause mortality during the malaria transmission season.^5^ Since then, numerous studies have been published documenting the health benefits of SMC in a range of settings, regimens, and age groups.

Since the WHO’s SMC policy recommendation in 2012, 13 countries in the Sahel subregion (Benin, Burkina Faso, Cameroon, Chad, The Gambia, Ghana, Guinea, Guinea–Bissau, Mali, Niger, Nigeria, Senegal, and Togo) have scaled up SMC, either nationally or sub-nationally. In 2015 and 2016, the Achieving Catalytic Expansion of SMC in the Sahel (ACCESS–SMC) supported the scale-up of SMC for 4 months per year among children under 5 years of age in seven Sahelian countries (Burkina Faso, Chad, The Gambia, Guinea, Mali, Niger, and Nigeria) and collected data on costs, feasibility, safety, and impact on malaria incidence and mortality, with a distribution of more than 25 million treatments to more than 7.5 million children in 2016.^6^ In 2021, almost 45 million children received at least one dose of SMC, and almost 180 million doses were delivered.^7^

Although the scale-up in providing up to four cycles of SMC to children 3–59 months old has provided millions of children with chemoprevention during malaria transmission season, many national malaria control programs are interested in increasing the number of rounds of treatment to cover a longer transmission season or extending the age range as the malaria burden shifts to older children. Previous systematic reviews of SMC among children younger than 5 years were published in 2011^5^ and 2012,^8^ finding reductions in clinical malaria of 82% and 74%, respectively. To potentially “include additional at-risk groups and clarify contextual considerations for implementation,” a WHO Chemoprevention Guidelines Development Group was convened, and an updated systematic review of the existing SMC evidence was requested.

We conducted a systematic review and meta-analyses of the impact of SMC on malaria disease burden among children at least 2 months of age (stratified by age range 2–59 months versus ≥ 60 months, drug regimen, and three or four versus five or six treatment cycles). We abstracted summary study-level data to evaluate the impact of SMC on the incidence of confirmed malaria, malaria prevalence, anemia prevalence, incidence of severe malaria, incidence of hospitalization for any cause, any-cause mortality, and adverse events to inform policy development.

## MATERIALS AND METHODS

The study protocol was reviewed and approved by the U.S. Centers for Disease Control and Prevention and by the WHO’s Guidelines Development Group on February 4, 2021.

### Search strategy.

The systematic review and meta-analyses adhered to Preferred Reporting Items for Systematic Reviews and Meta-Analyses (PRISMA) guidelines.^9^ The final search was conducted on March 2, 2021. We included all relevant studies for which data were available regardless of language or publication status. We searched MEDLINE (PubMed); EMBASE (OVID); PsycINFO (OVID); Global Health (OVID); Cochrane Library; CINAHL (EBSCOHost); Africa-Wide Information (EBSCOHost); Scopus; Global Index Medicus (WHO), which includes LILACS; and ClinicalTrials.gov. The following recent proceedings were reviewed for relevant abstracts: 7th Multilateral Initiative on Malaria Pan-African Malaria Conference (Dakar, Senegal; April 2018), American Society of Tropical Medicine and Hygiene (ASTMH) 67th Annual Meeting (New Orleans, LA; November 2018), ASTMH 68th Annual Meeting (National Harbor, MD; November 2019), and ASTMH 69th Annual Meeting (virtual meeting). We also searched the reference lists of previous reviews for previously unidentified studies.^5^^,^^8^^,^^10^^,^^11^ We contacted experts in the field to identify other studies or gray literature. Studies were identified through comprehensive electronic database searches using study-specific search terms (Supplemental File 1).

#### Eligible studies.

Randomized designs, including cluster-randomized controlled trials (cRCTs) with at least two clusters per arm, cluster-randomized stepped-wedge designs with at least four clusters, individually randomized controlled trials, and cluster-randomized cross-over trials with at least two clusters per arm and a suitable washout period, were eligible. In addition, nonrandomized designs including controlled before-and-after studies with a contemporaneous control group and at least two sites per arm and interrupted time series studies with at least two (with contemporaneous control group) or three (if no control group) data points both before the first round and after the last round of SMC, measured at evenly spaced intervals with a 1-year baseline, were eligible.

Participants included children > 2 months of age living in malaria-endemic areas of seasonal transmission. In this review, SMC was defined as administration of a full therapeutic course of antimalarial medicine (irrespective of the presence of symptoms or infection) to eligible children living in a defined geographic area (except those for whom the medicine was contraindicated) during the malaria transmission season, at approximately the same time and at repeated intervals. The comparator was standard of care. Although studies with malaria or non-malaria co-interventions were included, these had to be balanced in all arms. Studies also had to include one or more primary or secondary outcomes. The primary outcome was confirmed malaria illness incidence during the transmission season (starting from administration of the first cycle to 4–6 weeks after the last cycle), defined as febrile illness with diagnostically confirmed parasitemia and, owing to the geographic specificity, almost exclusively *P. falciparum*. Secondary outcomes were parasitemia prevalence measured through a cross-sectional survey at the end of the transmission season, 4–6 weeks after the last cycle (determined by microscopy, malaria rapid diagnostic test, or polymerase chain reaction); prevalence of moderate anemia (hemoglobin < 8 g/dL) measured through the same cross-sectional survey; all-cause hospital admissions; severe malaria (defined as the number of patients admitted with severe malaria); all-cause mortality; and adverse effects.

Studies were excluded if they did not take place in areas of seasonal transmission, did not administer at least three full treatment courses of antimalarials seasonally, included children < 2 months or > 15 years, did not have balanced co-interventions other than SMC, did not report one or more primary or secondary outcomes, or did not report primary data.

#### Study selection and data extraction.

**Figure 1.**
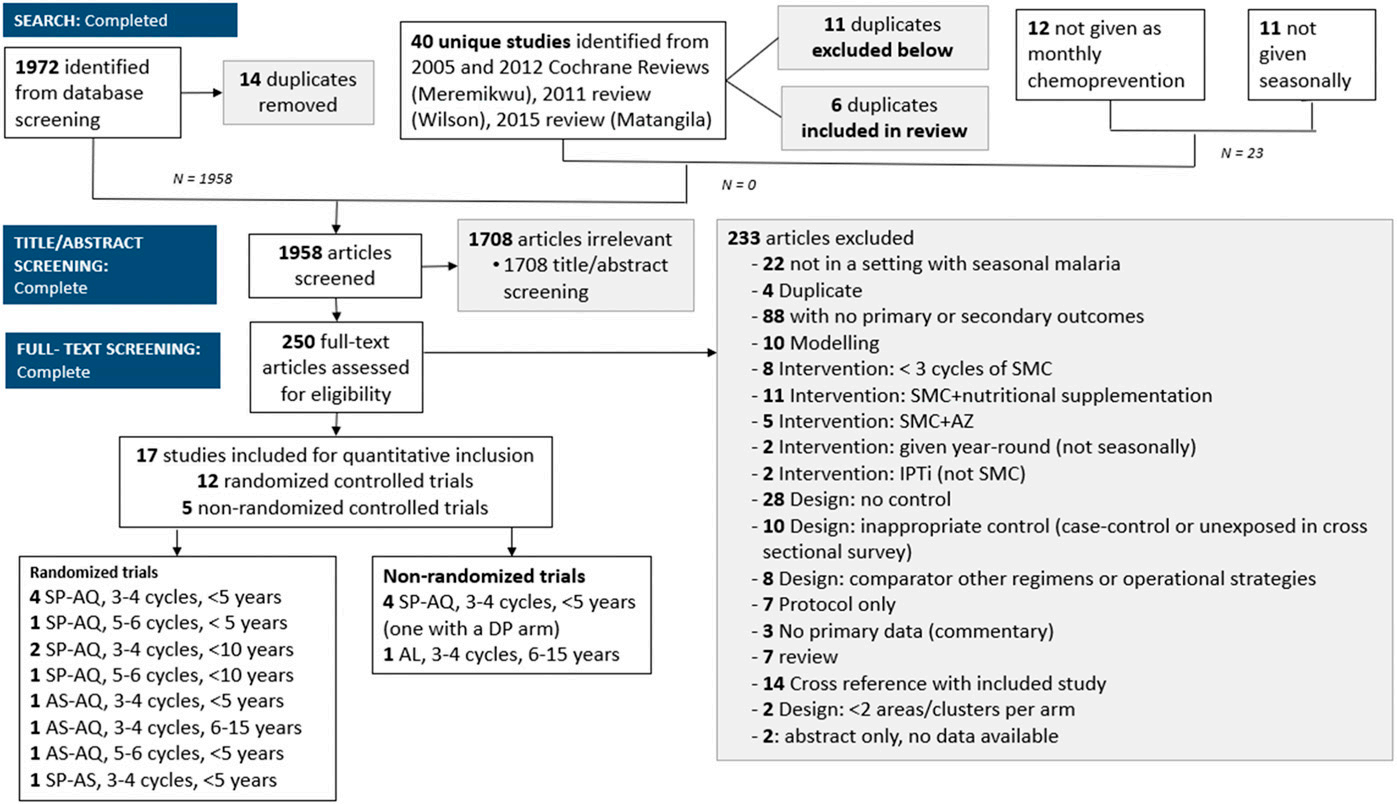
Preferred Reporting Items for Systematic Reviews and Meta-Analyses (PRISMA) diagram. AL = artemether–lumefantrine; AQ = amodiaquine; AS = artesunate; AZ = azithromycin; DP = dihydroartemisinin–piperaquine; IPTi = intermittent preventive treatment in infancy; SMC = seasonal malaria chemoprevention; SP = sulfadoxine–pyrimethamine.

**Figure 2.**
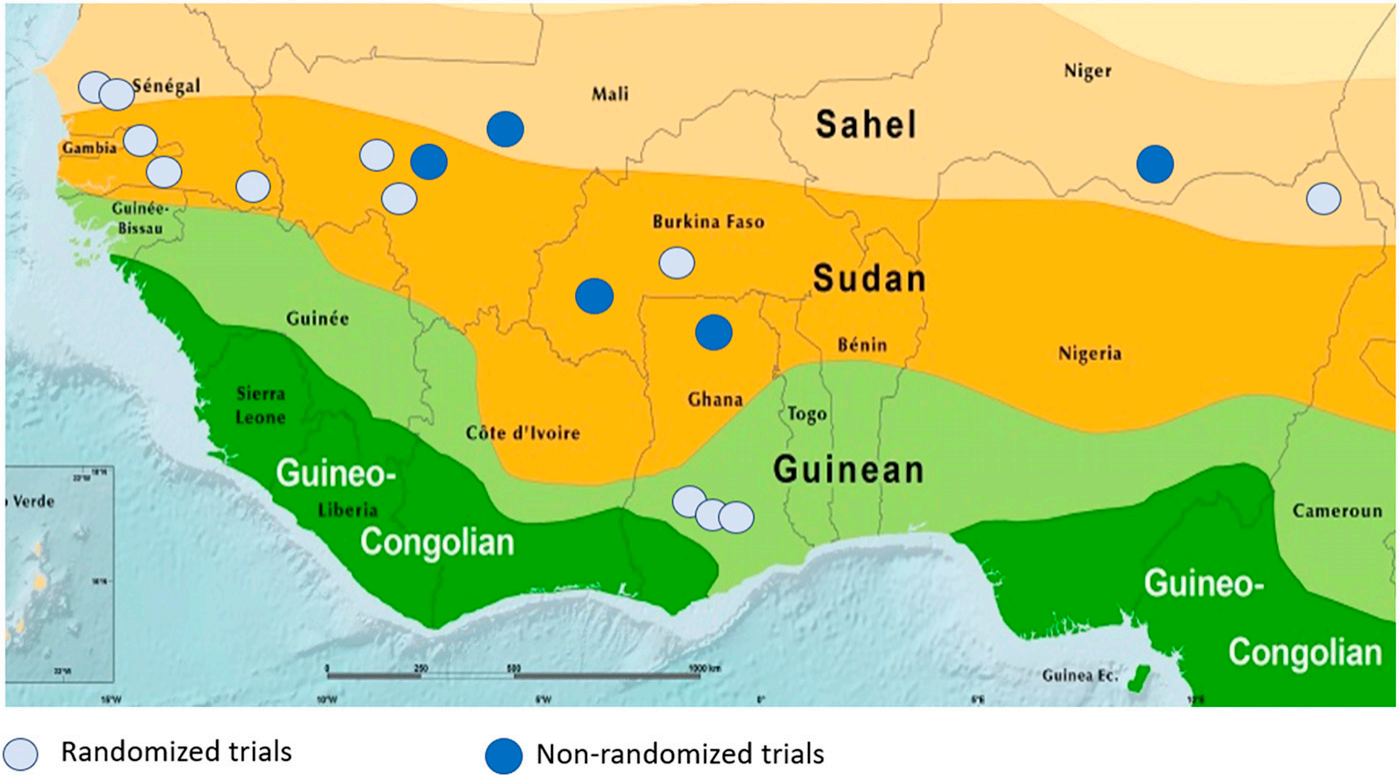
Location of eligible seasonal malaria chemoprevention studies. Zones: Sahel—mean annual precipitation 200–700 mm; Sudan—mean annual precipitation 700–900 mm; Guinean—mean annual precipitation 900–1,600 mm; and Guineo–Congolian—mean annual precipitation 1,600–2,000 mm.

Two reviewers independently screened titles and abstracts identified from literature searches based on the predetermined inclusion criteria and extracted information from selected studies on an electronic data extraction form. A third reviewer was consulted to resolve any disagreements. Publications reporting on the same study were grouped. Full-text studies that did not meet the eligibility criteria are listed with their reasons for exclusion in Supplemental File 2. The result of the study selection process is provided in a PRISMA flow diagram (Figure 1). Characteristics of included studies are included in Table 1. Locations of eligible SMC studies are included in Figure 2.

**Table 1:** Characteristics of included studies, randomized studies

Study	Country	Design	Age group	Comparison	No. of cycles	No. enrolled	Coverage	Parasite prevalence	ITN use	Rains	Months given
Ambe et al.^20^	Nigeria	cRCT	3–59 months	SP+AQ vs. placebo	4	204 SMC, 195 control	73% Received all cycles	79%	> 60%	Jun–Sept.	Aug., Sept., Oct., Nov.
Cissé et al.^21^	Senegal	iRCT	2–59 months	SP+AS vs. placebo	3	542 SMC, 546 control	∼95% per cycle	36–37%	< 25%	July–Sept.	Sept., Oct., Nov.
Cissé et al.^22^	Senegal	Stepped wedge	3–119 months	SP+AQ vs. nothing	3	2008: 14,000 2009: 90,000 2010: 160,000	2008: 93% 2009: 84% 2010: 93%	1–2%	50–80%	July–Sept.	Sept., Oct., Nov.
Dicko et al.^23^	Mali	iRCT	3–59 months	SP+AQ vs. placebo	3	1,509 SMC, 1,508 placebo	> 95% per cycle	13%	> 90%	July–Oct.	Aug., Sept., Oct.
Konate et al.^24^	Burkina Faso	iRCT	3–59 months	SP+AQ vs. placebo	3	1,509 SMC, 1,505 placebo	70–80% per cycle	42%	> 90%	July–Oct.	Aug., Sept., Oct.
Kweku et al.^25^	Ghana	iRCT	3–59 months	AS–AQ vs. placebo	6	626 SMC, 650 placebo	> 95% per cycle	20%	< 25%	April–July, Sept.–Nov.	May, June, July, Aug., Sept., Oct.
Ndiaye et al.^26^	Senegal	cRCT	3–119 months	SP+AQ vs. nothing	5	2,245 SMC, 2,301 control	> 90% per cycle	18% < 5 years; 25% 5–9 years	> 90%	May–Oct.	July, Aug., Sept., Oct., Nov.
Sesay et al.^27^	The Gambia	iRCT	6–59 months	SP+AQ vs. placebo	3	639 SMC, 638 placebo	> 95% per cycle	1%	> 90%	July–Sept.	Sept., Oct., Nov.
Tagbor et al.^28^	Ghana	cRCT	3–59 months	AS–AQ vs. nothing	3	800 SMC, 800 control	30% Received all cycles	33%	NR	May–Nov.	May, July, Sept.
Tagbor et al.^29^	Ghana	iRCT	3–59 months	SP+AQ vs. placebo	5	741 SMC, 749 control	36% Received all cycles; ∼70% per cycle	27%	> 90%	May–Nov.	July, Aug., Sept., Oct., Nov.
Thera et al.^30^	Mali	iRCT	6–15 months	AS–AQ vs. nothing	4	100 SMC, 100 control	NR	8–15%	NR	July–Oct.	Oct., Nov., Dec., Jan.
Tine et al.^31^	Senegal	cRCT	3–119 months	SP+AQ vs. nothing	3	1,000; 500 per arm	∼90% per cycle	9–11%	> 90%	July–Oct.	Sept., Oct., Nov.

AQ = amodiaquine; cRCT = cluster-randomized controlled trial; iRCT = individually randomized controlled trial; ITN = insecticide treated net; NR = not reported, SMC = seasonal malaria chemoprevention; SP = sulfadoxine–pyrimethamine.

For dichotomous outcomes (prevalence outcomes), we extracted the number of events and number of participants in each study arm. For rate data (incidence and mortality), we extracted the number of events, the total person–time at risk or population at risk in each study arm, and a measure of variance (standard error) when available or rate ratio when reported. Among studies that administered SMC to children up to 10 years of age, where these were presented separately, we abstracted and presented results by children < 5 years and children ≥ 5 years.

Incidence measures (incidence, severe malaria, hospitalization, and death) were reported starting from the day of the first cycle to 4–6 weeks after the last cycle in both arms, whereas prevalence measures (parasitemia, anemia) were a comparison between arms of an endline cross-sectional survey conducted within 4–6 weeks after the last cycle. In studies in which a baseline cross-sectional survey was performed, it was conducted 1–2 months before the first cycle of SMC, and when a baseline survey was reported in addition to an endline, we performed a difference-in-differences (DiD) analysis. Although some studies performed cross-sectional surveys a year after the end of the last SMC cycle or included incidence measures through the season after SMC, these were performed to evaluate rebound phenomena, which were not included in this analysis.

Potential effect modifiers including transmission intensity (incidence or prevalence), age, choice of drug, cycles per year, months of rain, timing (in relation to season) of administration, and coverage of vector control were collected when available (though not included in the meta-analysis given the small number of studies).

**Figure 3.**
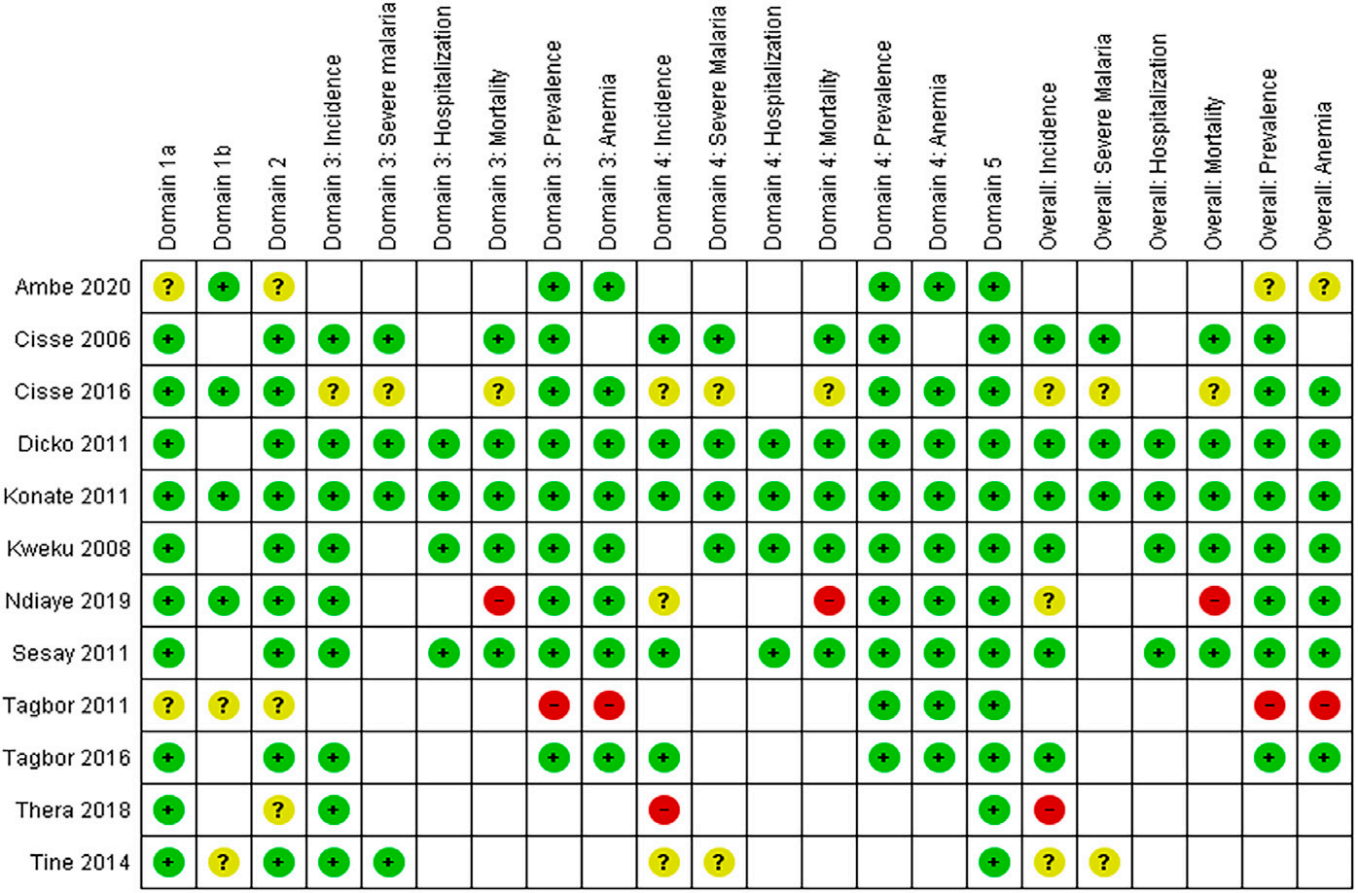
Risk of bias summary: review authors’ judgments about each risk of bias item for each included study by malaria outcomes, randomized studies. Revised risk of bias (RoB 2) tool: Domain 1a. Risk of bias arising from randomization; Domain 1b. Risk of bias from timing of identification/recruitment; Domain 2. Risk of bias due to deviations from intended intervention; Domain 3. Missing outcome data; Domain 4. Risk of bias in measurement of the outcome; Domain 5. Risk of bias in selection of reported result. Color code: green—low risk of bias; yellow—some concern for bias; red—high risk of bias.

Two members of the review team independently assessed the risk of bias for each study and each outcome. Any discrepancies were resolved through discussion. The Revised Cochrane “Risk of Bias” (RoB 2) tool^12^ from the *Cochrane Handbook for Systematic Reviews of Interventions* was used for individual randomized studies, and the RoB 2 Cluster-Randomized Trial (CRT) worksheet was used for CRTs.^13^ We assessed nonrandomized controlled studies using the Cochrane Risk of Bias In Non‐randomized Studies – of Interventions (ROBINS‐I) tool,^14^ which recommends including only nonrandomized studies that are not classified as having critical risk of bias. Risk of bias in selected studies is reported in Figure 3.

Given the critical risk of bias found in the nonrandomized studies and the number of high-quality randomized trials available, we included only randomized studies in the primary analysis. The nonrandomized trials are presented in Supplemental File 3.

### Data synthesis and meta-analysis.

Risk ratios and prevalence ratios were used to summarize treatment group differences for dichotomous outcomes for studies with a control group, and rate ratios correspondingly were used for count outcomes. Measures of effects were presented with corresponding 95% CIs. To calculate the incidence (of confirmed malaria, severe malaria, all-cause hospitalizations, and all-cause mortality), the denominator was the person–time of observation calculated as total months of observation across the study group or observed population. When controlled before-and-after measures for prevalence (of parasitemia and anemia) were presented, we abstracted numerators and denominators for each arm before and after and performed a DiD analysis. For cluster RCTs, measures of effect adjusted for clustering were used when possible. We conducted the meta-analyses and constructed forest plots using ReviewManager 5.

We stratified first by age group (< 5 years versus ≥ 5 years), by drug regimen (SP+AQ) versus artesunate-amodiaquine (AS-AQ) versus sulfadoxine–pyrimethamine + artesunate (SP+AS), and by number of monthly cycles (three or four cycles versus five or six cycles). Only randomized studies were included in the meta-analyses. For each study, a risk ratio or rate ratio was calculated or abstracted, log[RR] and standard error were calculated, and inverse variance weighting was used in the meta-analysis. We assessed heterogeneity by examining forest plots for overlapping CIs. Statistical heterogeneity was examined using the *I*^2^ statistic and classified according to the Cochrane Handbook criteria: moderate: *I*^2^ values 30–60%; substantial: *I*^2^ values 50–90%; and considerable: *I*^2^ values 75–100%. Given the small number of studies included in each meta-analysis, we used a fixed-effects meta-analysis. There were insufficient numbers of studies included in each meta-analysis to evaluate for reporting bias.

## RESULTS

Of the 2,012 titles identified by the search strategy, 250 underwent full-text review, and 12 randomized and five nonrandomized studies met the inclusion criteria (Figure 1). Results for nonrandomized studies are presented in Supplemental File 3.^15^^–^^19^

The randomized studies were conducted in six countries across the Sahel: Burkina Faso,^24^ The Gambia,^27^ Ghana,^25^^,^^28^^,^^29^ Mali,^23^^,^^30^ Nigeria,^20^ and Senegal^21^^,^^22^^,^^26^^,^^31^ (Table 1). Four were cluster-randomized studies, seven were individually randomized studies, and one was a randomized stepped-wedge study. Six used three or four cycles of SP+AQ (four including only children < 5 years of age and two including children < 10 years of age), and two used five or six cycles of SP+AQ (one including children < 5 years of age and one including children < 10 years of age). Of the remaining four, one used three or four cycles of AS–AQ among schoolchildren 6–15 years of age, and one each used three or four cycles of AS–AQ, five or six cycles of AS–AQ, and three or four cycles of SP+AS, all among children under 5 years. Parasite prevalence at baseline ranged from 1% in Senegal and The Gambia to 79% in Nigeria. Although most studies achieved relatively high coverage (∼90% per cycle), two studies performed in Ghana^28^^,^^29^ reported approximately 70% coverage per cycle, with < 40% of children having received all targeted cycles.

**Figure 4.**
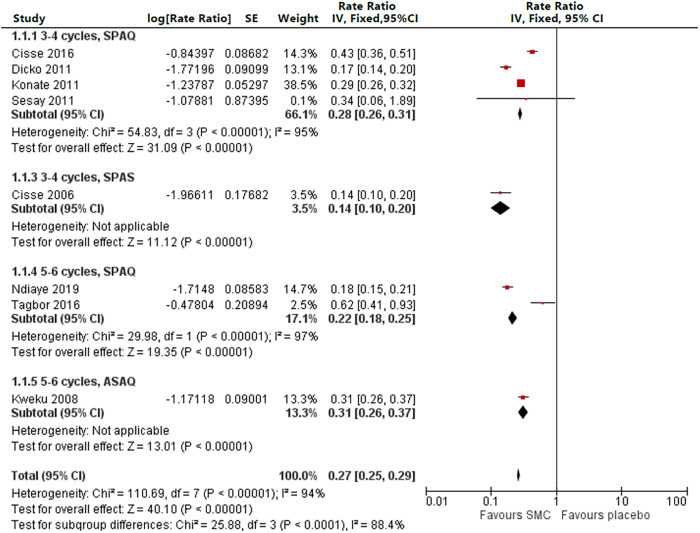
Incidence among children < 5 years of age by drug regimen, three or four cycles versus five or six cycles. AS–AQ = artesunate–amodiaquine; df = degrees of freedom; SE = standard error; SMC = seasonal malaria chemoprevention; SP+AQ = sulfadoxine–pyrimethamine + amodiaquine; SP+AS = sulfadoxine–pyrimethamine + artesunate.

Eight studies reported an impact on confirmed malaria incidence during the transmission season among children < 5 years of age (four with three or four cycles of SP+AQ,^22^^–^^24^^,^^27^ one with three or four cycles of SP–AS,^21^ two with five or six cycles of SP+AQ,^26^^,^^29^ and one with five or six cycles of AS–AQ,^25^ with rate ratios ranging 0.14–0.62, an overall rate ratio of 0.27 [95% CI: 0.25–0.29], and an *I*^2^ of 94%, demonstrating considerable heterogeneity). However, with the exception of the study with a baseline parasite prevalence of 1%,^27^ all studies demonstrated a significant reduction in incidence, and with the exception of a study with low coverage (36% received all cycles),^29^ all rate ratios were < 0.45 (Figure 4).

**Figure 5.**
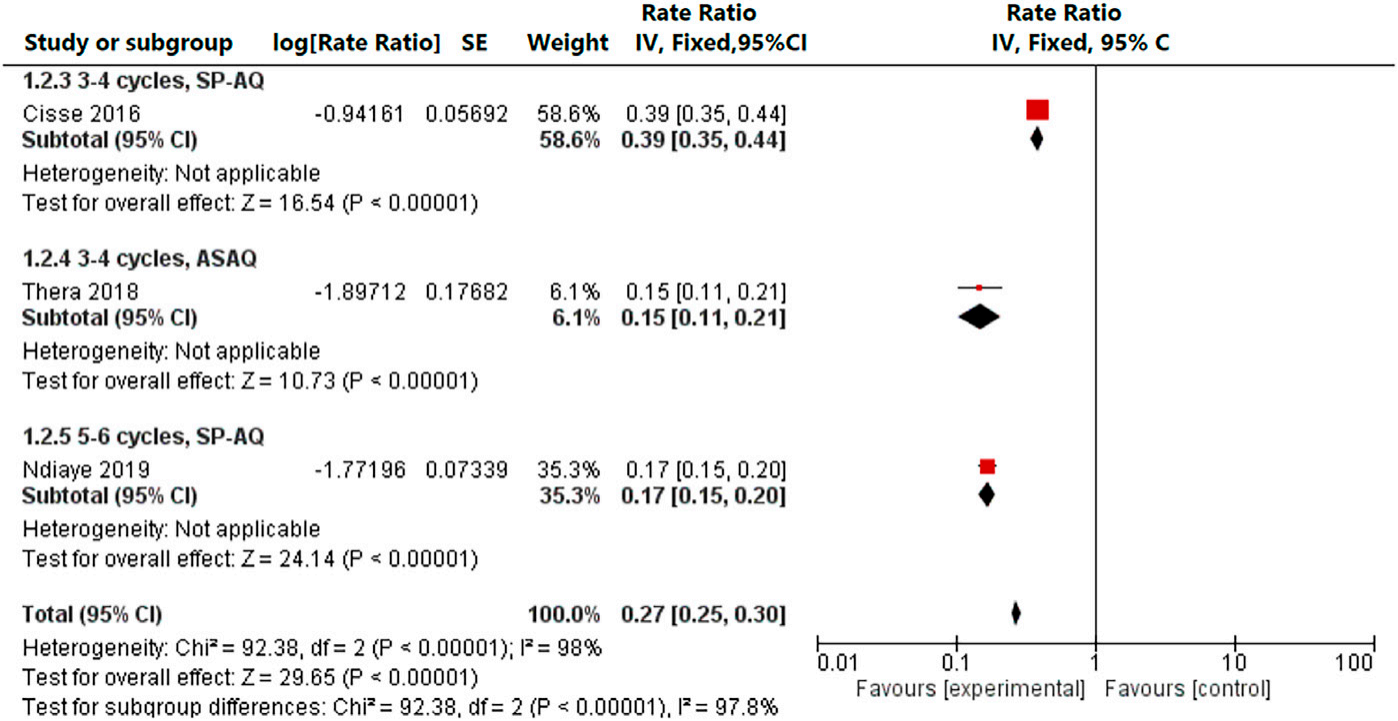
Incidence among children ≥ 5 years of age by drug regimen, three or four cycles versus five or six cycles, randomized studies. AS–AQ = artesunate–amodiaquine; SE = standard error; SP+AQ = sulfadoxine–pyrimethamine + amodiaquine.

Three studies reported an impact on malaria incidence during the transmission season among children ≥ 5 years, one with three or four cycles of SP+AQ,^22^ one with five or six cycles of SP+AQ,^26^ and one with three or four cycles of AS–AQ in schoolchildren aged 6–15 years,^30^ with rate ratios of 0.15–0.39, an overall rate ratio of 0.27 (0.25–0.30), and an *I*^2^ of 98%, demonstrating considerable heterogeneity (Figure 5). Three studies in Senegal reported an incidence for children < 10 years of age; two used three cycles of SP+AQ,^22^^,^^31^ whereas one used five cycles of SP+AQ.^26^ In this comparison, three cycles were associated with a rate ratio of 0.40 (95% CI: 0.35–0.45; *I*^2^ = 0%) compared with a rate ratio of 0.17 (95% CI: 0.15–0.20) for five cycles. Among the two studies that reported children < 5 years of age and children ≥ 5 years separately,^22^^,^^26^ there was no difference in risk reduction between children < 5 years and children ≥ 5 years (*P* = 0.35^22^ and *P* = 0.61,^26^ respectively).

**Figure 6.**
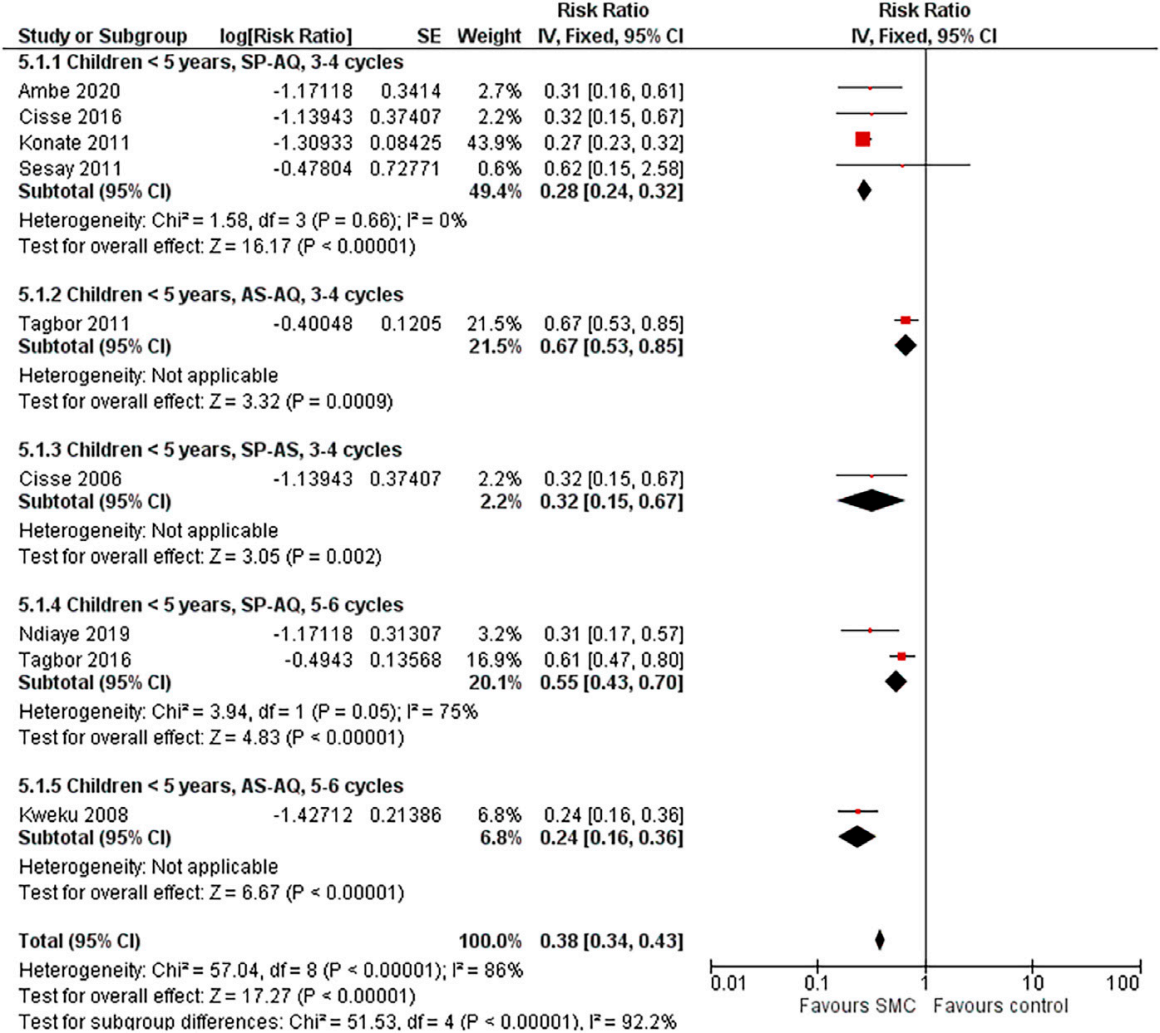
Prevalence among children < 5 years of age by drug regimen, three or four cycles versus five or six cycles, randomized studies. AS–AQ = artesunate–amodiaquine; SE = standard error; SMC = seasonal malaria chemoprevention; SP+AQ = sulfadoxine–pyrimethamine + amodiaquine; SP+AS = sulfadoxine–pyrimethamine + artesunate.

Nine studies reported malaria prevalence at the end of the transmission season among children < 5 years of age (four with three or four cycles of SP+AQ,^20^^,^^22^^,^^24^^,^^27^ one with three or four cycles of AS–AQ,^28^ one with three or four cycles of SP–AS,^21^ two with five or six cycles of SP+AQ,^26^^,^^29^ and one with five or six cycles of AS–AQ^25^), with a range of risk ratios 0.24–0.67 and an overall risk ratio of 0.38 (95% CI: 0.34–0.43; *I*^2^ = 86%). When the study with a baseline parasite prevalence of 1%^27^ and the two studies with low coverage^28^^,^^29^ were removed, the range of risk ratios was 0.24–0.32 (Figure 6). Two studies in Senegal reported malaria prevalence among children ≥ 5 years: One study reported malaria prevalence separately among children 5–9 years of age,^26^ with a risk ratio of 0.23 (95% CI: 0.11–0.48), and two studies reported prevalence among all children < 10 years,^22^^,^^31^ with an overall risk ratio of 0.28 (95% CI: 0.17–0.44; *I*^2^ = 43%).

The prevalence of any anemia (hemoglobin < 11 g/dL) at the end of the transmission season among children < 5 years of age was reported by six studies. The three studies of three or four cycles of SP+AQ in high-transmission zones (baseline parasite prevalence > 35%)^20^^,^^23^^,^^24^ demonstrated a moderate effect size (risk ratio: 0.77, 95% CI: 0.72–0.83, *I*^2^ = 87%), and one study of five or six cycles of SP+AQ demonstrated a small effect size (risk ratio: 0.88, 95% CI: 0.88–0.97).^26^ Two additional studies performed in Ghana (one with three or four cycles of AS–AQ and one with five or six cycles of SP+AQ) that had low coverage (< 80% per cycle or < 40% who received all cycles) did not demonstrate a protective effect.^28^^,^^29^ One study reported the prevalence of any anemia among children ≥ 5 years and demonstrated a moderate protective effect (risk ratio: 0.70, 95% CI: 0.52–0.95).^26^

Six studies also reported the prevalence of moderate anemia at the end of the transmission season (hemoglobin < 8 g/dL) among children < 5 years of age. Two studies in zones of low transmission (baseline parasite prevalence < 10%) did not show a protective effect (risk ratio: 0.93, 95% CI: 0.81–1.07, *I*^2^ = 0%).^22^^,^^27^ Two studies in zones of moderate to high transmission (baseline parasite prevalence ≥ 10%) showed a moderate reduction in moderate anemia (risk ratio: 0.47, 95% CI: 0.35–0.63, *I*^2^ = 0%).^23^^,^^24^ A study of five or six cycles of AS–AQ (risk ratio: 0.91, 95% CI: 0.64–1.30) and a study of five or six cycles of SP+AQ (risk ratio: 0.67, 95% CI: 0.30–1.48) did not show a reduction.^25^^,^^29^ Two studies from Senegal^22^^,^^26^ reported a prevalence of severe anemia (hemoglobin < 5 g/dL), but the extremely low prevalence resulted in wide CIs and nonsignificant reductions.

Three studies reported the incidence of severe malaria during the transmission season among children < 5 years of age receiving three or four cycles of SP+AQ,^22^^–^^24^ and one study reported the incidence of severe malaria among children 5–9 years of age receiving three or four cycles of SP+AQ.^22^ These studies demonstrated a moderate reduction in severe malaria across transmission intensities, with an overall rate ratio of 0.53 (95% CI: 0.37–0.76, *I*^2^ = 30%). Stratified by age, among children < 5 years the rate ratio was 0.57 (95% CI: 0.37–0.89), and among children 5–9 years of age (one study), the rate ratio was 0.44 (95% CI: 0.23–0.84).

Four studies reported hospitalization during the transmission season for any cause among children < 5 years. Two studies of three or four cycles of SP+AQ in zones of low to moderate transmission did not show a reduction in all-cause hospitalization (rate ratio: 1.38, 95% CI: 0.71–2.67, *I*^2^ = 0%).^23^^,^^27^ However, two studies in higher transmission zones showed a reduction: a study of three or four cycles of SP+AQ (rate ratio: 0.54, 95% CI: 0.31–0.94)^24^ and a study of five or six cycles of AS–AQ (rate ratio: 0.42, 95% CI: 0.20–0.87).^25^

Six studies reported all-cause mortality among children < 5 years of age, four studies among children receiving three or four cycles of SP+AQ,^22^^–^^24^^,^^27^ one study among children < 5 years of age receiving five or six cycles of SP+AQ,^26^ and one study among children receiving five or six cycles of AS–AQ.^25^ There was no mortality reduction, with an overall rate ratio of 0.89 (95% CI: 0.68–1.17, *I*^2^ = 0%). Two studies reported all-cause mortality among children ≥ 5 years, one study among children receiving three or four cycles of SP+AQ^22^ and one study among children receiving five or six cycles of SP+AQ.^26^ Similarly, there was no mortality reduction, with an overall rate ratio of 0.99 (95% CI: 0.62–1.59, *I*^2^ = 0%).

No severe adverse reactions related to the intervention were reported in the included studies. Only Cissé et al.,^22^ which administered a total of 776,191 documented cycles over 3 years to children up to 120 months of age, reported five serious adverse events possibly related to the intervention (Table 2). An independent review panel considered that only extrapyramidal syndrome was likely to be related to study drugs.^22^ Of the 11 studies that documented adverse events, there were a variety of methodologies (active versus passive versus enhanced passive, only in intervention arms, etc.). Four individually randomized studies^21^^,^^23^^,^^24^^,^^30^ conducted active surveillance and reported events in both intervention and placebo arms, all among children receiving three or four doses of SP+AQ (three studies among children < 5 years of age and one among schoolchildren 6*–*14 years of age). Mild to moderate adverse events were increased in the intervention arm (risk ratio: 1.40, 95% CI: 1.31*–*1.51, *I*^2^ = 0%), the vast majority nausea/vomiting, abdominal pain, and headache. No other adverse events were significantly higher in the intervention arm.

**Table 2:** Serious adverse events Cissé et al.^22^

Patient	Age	Adverse event	Timing
1	9 years	Acute diarrheal disease leading to death	1 week after first cycle
2	9 years	Rash and facial edema	2 days after first cycle
3	5 years	Jaundice (yellow sclera, no liver tests done)*	2 days after second cycle
4	8 years	Extrapyramidal syndrome	2 days after first cycle
5	17 months	Skin rash (detected through active surveillance)†	2 weeks after first cycle

* Child also took other medicines, including acetaminophen.

† Dermatologists determined likely staphylococcal infection.

## DISCUSSION

Regardless of transmission setting or number of cycles, SMC resulted in substantial reductions in malaria incidence and prevalence and moderate reductions in severe malaria and prevalence of any anemia among both children < 5 years of age and children ≥ 5 years. In zones of moderate to high transmission, SMC resulted in reductions in moderate anemia and all-cause hospitalization among children < 5 years of age. No mortality reduction was detected. Although four of the 12 studies included had drug regimens including artesunate (SP+AS, AS–AQ) and some achieved results comparable to those using SP+AQ, the use of artesunate in a chemopreventive regimen is strongly discouraged, given the emerging threat of artemisinin resistance and the need to safeguard its use as treatment for as long as possible. In practice, these findings pertain to SMC using SP+AQ.

Effect sizes were similar for children < 5 years of age and children ≥ 5 years for the incidence of uncomplicated and severe malaria during the transmission season and for malaria prevalence and prevalence of any anemia at the end of the transmission season, suggesting that children ≥ 5 years may benefit from SMC as much as children < 5 years. Although one study in Mali was conducted among schoolchildren aged 6–14 years (and reported only incidence and adverse events), the other three studies (reporting the majority of outcomes) among children < 10 years of age were all conducted in Senegal, with lower malaria prevalence than in most studies, even in Senegal’s high-transmission southeast.

No study directly compared outcomes between children who received three or four cycles and children who received five or six cycles of SMC; studies of five or six cycles of SMC were conducted where transmission was longer than 4 months (three studies). One study in Ghana (SP+AQ) reported low coverage (approximately 70% received each round),^29^ but the other two studies, SP+AQ in Senegal^26^ and AS–AQ in Ghana,^25^ which reported > 90% coverage, showed very similar risk reductions to trials of three or four cycles.

Although 12 randomized studies were included in the analysis, there was substantial heterogeneity because of the different combinations of age range, drug regimen, number of cycles, coverage of other interventions, and variability in transmission intensity. Nonetheless, effect sizes were surprisingly consistent. Because interventions other than SMC were required to be balanced in both arms, a number of strategies in which SMC administration was combined with another intervention were ineligible for inclusion: SMC combined with azithromycin administration,^32^ SMC combined with micronutrient powders or other nutritional interventions,^33^^–^^35^ or SMC in combination with distribution of long-lasting insecticide-treated nets.^36^ Other studies have directly compared SMC delivery methods^37^^–^^39^ or drug regimens.^40^^,^^41^ Innovations currently under study include administration of all three doses of each cycle under directly observed therapy.

It should be noted that every study eligible for inclusion was performed in the Sahel, where high-grade resistance to SP remains relatively low. Although the prevalence of *pfdhfr* mutations associated with resistance to pyrimethamine (including the triple mutant *pfdhfr* S108N, C59R, N51I) is high in the Sahel, *pfdhps* mutations, in particular the triple mutant *pfdhps* A437G, K540E, A581G, are rare.^42^ The efficacy of chemoprevention with SP alone has been shown to be minimal in areas of southern and eastern Africa, with > 50% prevalence of quintuple mutants (*pfdhfr* S108N, C59R, N51I and *pfdhps* A437G, K540E).^42^^,^^43^ Ongoing efficacy of the AQ component is also important; the duration of chemoprevention of regimens containing AQ is decreased when mutations associated with AQ resistance (*pfmdr1* N86Y and *pfcrt* K76T) are present at > 80% prevalence^44^; thus, it is paramount to ensure that treatment regimens do not contain AQ in areas in which SMC is implemented, which would exacerbate drug pressure on the AQ component. Close monitoring of indicators of resistance to SP and AQ is warranted in areas in which SMC is implemented.

In comparison to this review, a review published in 2011 included seven controlled and five uncontrolled studies and found an 82% reduction in clinical malaria among controlled studies,^5^ whereas a review published in 2012 that included seven studies (six of which were the same as the controlled studies in the 2011 review) found a 74% reduction in clinical malaria incidence.^8^ This meta-analysis, which included only randomized controlled trials, incorporated six studies included in a previous review and added six studies published since the 2012 review, several of which included additional cycles or extended age eligibility; nevertheless, it found an almost identical 73% reduction (rate ratio: 0.27) in the incidence of uncomplicated malaria, both for children aged < 5 years and children ≥ 5 years.

The ACCESS–SMC Partnership published information on SMC scale-up in 2015 and 2016 in Burkina Faso, Chad, The Gambia, Guinea, Mali, Niger, and Nigeria, including a case-control study to monitor protective efficacy in the programmatic setting. In data collected from Burkina Faso in 2016, Chad in 2016, The Gambia in 2015 and 2016, Mali in 2015 and 2016, and Nigeria in 2016, among 2,185 confirmed cases and 4,370 controls, a random effects meta-analysis found a protective effectiveness against clinical malaria incidence of 88% (pooled odds ratio [OR]: 0.12, 95% CI: 0.06–0.21) in the first 28 days after drug administration and 61% (pooled OR: 0.39, 95% CI: 0.28–0.53) in days 29–42 after drug administration.^45^^,^^46^

Programmatic reports from implementation have also included serious and severe adverse events captured by the health system. During the first 3 years of SMC (2014–2016), covering over 5 million cycles, Senegal reported one case of Lyell syndrome, two cases of Stevens–Johnson syndrome, one case of extrapyramidal syndrome, and three allergic reactions.^47^ The ACCESS–SMC Partnership reported 36 serious adverse events in more than 37.5 million cycles in 2015 and 2016, including one rash, two fevers, one extrapyramidal syndrome, one Quincke edema, and 31 gastrointestinal conditions.^45^

After the literature search and data abstraction were completed, Chandramohan et al.^48^ published the results of a study comparing SMC with the RTS,S vaccine with SMC+RTS,S. In this three-arm, individually randomized controlled trial, 6,861 children aged 5–17 months were randomized to SP+AQ (2,287), RTS,S (2,288), or SMC+ RTS,S (2,286). Of these, 1965, 1988, and 1967, respectively, received the first dose and were followed up for 3 years. The incidence of malaria was 305 per 1,000 person–years in the SMC arm, 278 per 1,000 person–years in the RTS,S arm, and 113 per 1,000 person–years in the SMC+RTS,S arm. The combination of SMC and RTS,S was 59.6% (95% CI: 54.7–64.0%) more effective against clinical malaria, 70.6% (95% CI: 42.3–85.0%) more effective against hospital admission with severe malaria, and 75.3% (95% CI: 12.5–93.0%) more effective against mortality due to malaria than RTS,S alone.^48^

## CONCLUSION

Seasonal malaria chemoprevention is highly effective at reducing malaria disease burden among children who receive the intervention, and evidence suggests that expanding the age range beyond 5 years and for longer than four cycles may be appropriate in some settings. The WHO has introduced an online platform that allows users to access the most up-to-date recommendations (https://app.magicapp.org/#/guideline/LwRMXj/section/EPMOYj), including for SMC. Although SMC has been recommended for zones of seasonal transmission where there is not substantial parasite resistance to SP, studies are ongoing in the belt of seasonal transmission in southern Africa to assess the suitability of the intervention where resistance to SP is substantial. Other reviewers are examining contextual factors such as implementation strategies, feasibility, cost-effectiveness, and equity. Although evidence to date supports expanding age ranges and number of cycles as countries pilot or scale these interventions, further evidence of impact and safety would be beneficial.

## Supplemental files

10.4269/ajtmh.23-0481 Supplemental Materials
