# Supplementary material for: Systematic Review and Meta-Analysis of Seasonal Malaria Chemoprevention
Source: Am J Trop Med Hyg. 2023 Dec 11;110(1):20–31. doi: 10.4269/ajtmh.23-0481 (PMC10793029; doi:10.4269/ajtmh.23-0481)
Supplement: Supplemental Materials [file tpmd230481.SD1.pdf]

Search Query: Systematic review on seasonal malaria chemoprevention/chemoprophylaxis AND seasonal intermittent preventive treatment of malaria in children. Exclude pregnant/pregnancy.

Search References:
Meremikwu MM, Donegan S, Sinclair D, Esu E, Oringanje C. Intermittent preventive treatment for malaria in children living in areas with seasonal transmission. Cochrane Database Syst Rev. 2012 Feb 15;2012(2):CD003756.

Search Strategy:

Database	Strategy	Run Date	Records
**Medline (OVID) 1946-**	Malaria* OR antimalaria* OR anti-malaria*<br>AND<br>Chemoprevention OR chemoprophylaxis OR Chemo-prevention OR chemo-prophylaxis OR intermittent preventive<br>AND<br>Child* OR school* OR preschool* OR infant* OR newborn* OR neonat*<br>Limit 2000 – ;	3/2/2021	285
**Embase (OVID) 1988-**	Malaria* OR antimalaria* OR anti-malaria*<br>AND<br>Chemoprevention OR chemoprophylaxis OR Chemo-prevention OR chemo-prophylaxis OR intermittent preventive<br>AND<br>Child* OR school* OR preschool* OR infant* OR newborn* OR neonat*<br>Not pubmed/medline<br>Limit 2000 – ;	3/2/2021	327<br>-179 duplicates<br>=148 unique items
**PsycINFO (OVID) 1806-**	Malaria* OR antimalaria* OR anti-malaria*<br>AND<br>Chemoprevention OR chemoprophylaxis OR Chemo-prevention OR chemo-prophylaxis OR intermittent preventive<br>AND<br>Child* OR school* OR preschool* OR infant* OR newborn* OR neonat*<br>Limit 2000 – ;	3/2/2021	2<br>-1 duplicates<br>=1 unique items
**Global Health (OVID) 1910-**	Malaria* OR antimalaria* OR anti-malaria*<br>AND<br>Chemoprevention OR chemoprophylaxis OR Chemo-prevention OR chemo-prophylaxis OR intermittent preventive<br>AND<br>Child* OR school* OR preschool* OR infant* OR newborn* OR neonat*<br>Limit 2000 – ;	3/2/2021	268<br>-191 duplicates<br>=77 unique items
**Cochrane Library**	Malaria* OR antimalaria* OR anti-malaria*<br>AND	3/2/2021	60<br>-51

Supplemental File 2: Excluded studies

Study	Title	Reason for exclusion
Abiola 2015	Impact of seasonal malaria chemoprevention in the production of msp1 the ama1 antibodies in Senegal	no outcomes
Ahorlu 2009	Effectiveness of intermittent preventive treatment for children (IPTC) combined with timely treatment at home for malaria control	intervention: year round
Ahorlu 2009	Effectiveness of combined intermittent preventive treatment for children and timely home treatment for malaria control	intervention: year round
Ahorlu 2011	Two-year evaluation of Intermittent Preventive Treatment for Children (IPTc) combined with timely home treatment for malaria control in Ghana	duplicate
Aikins 2017	COST-EFFECTIVENESS OF SEASONAL MALARIA CHEMOPREVENTION IN UPPER WEST REGION OF GHANA	no outcomes
Alexander 2007	Modelling the impact of intermittent preventive treatment for malaria on selection pressure for drug resistance	modeling
Angoran-Benie 2019	Community-based approach to reach malnourished infants from 6 months to 5 years during a seasonal malaria chemoprevention (SMC) campaign in remote areas in Niger	intervention: SMC+nutrition
Anne 2018	Strategy for improving the treatment of seasonal malaria chemoprevention among children from 3 to 120 months of age in Goudomp health district, Senegal: A 3-day directly observed treatment initiative	no outcomes
Anne 2019	Seasonal malaria chimio prevention 2017 in the health district of Goudomp Senegal cost-effectiveness analysis of two treatment strategies for children aged 3-120 months	no outcomes
Anonymous 2016	Corrigendum: Impact of combined intermittent preventive treatment of malaria and helminths on anaemia, sustained attention, and recall in Northern Ghanaian schoolchildren	correction
Anonymous 2016	Correction: safety of Seasonal Malaria Chemoprevention (SMC) with sulfadoxine-pyrimethamine plus amodiaquine when delivered to children under 10 years of age by district health services in Senegal: results from a stepped-wedge cluster randomized trial (PLoS ONE (2016) 11: 10 (e0162563) DOI: 10.1371/journal.pone.0162563)	link to included
Ansah 2016	Evaluation of the impact of seasonal malaria chemoprevention on mortality and mortality in young children in Northern Ghana	design: one cluster per arm
Ansah 2016	Evaluation of the impact of implementation of seasonal malaria chemoprevention on morbidity and mortality in young children: A qualitative study in Northern Ghana	no outcomes
Antwi 2016	Facilitators and Barriers to Uptake of an Extended Seasonal Malaria Chemoprevention Programme in Ghana: A Qualitative Study of Caregivers and Community Health Workers	no outcomes
Attaher 2016	Influence of seasonal malaria chemoprevention on markers of T cell exhaustion and immunoregulation	no outcomes
Attaher 2020	Effect of Seasonal Malaria Chemoprevention on Immune Markers of Exhaustion and Regulation	no outcomes
Ba 2014	Extending the age range for seasonal malaria chemoprevention (SMC): Effectiveness of SMC in children under 10 years of age delivered through the district health service in Senegal	link to included
Ba 2018	Implementation, coverage and equity of large-scale door-to-door delivery of Seasonal Malaria Chemoprevention (SMC) to children under 10 in Senegal	no outcomes

Barger 2009	Intermittent preventive treatment using artemisinin-based combination therapy reduces malaria morbidity among school-aged children in Mali	intervention: < 3 cycles
Barry 2016	Comparison of three versus four rounds of seasonal malaria chemoprevention on the incidence of clinical malaria in Mali	no control - compares 3 vs 4 cycles
Barry 2018	Optimal mode for delivery of seasonal malaria chemoprevention in Ouelessebougou, Mali: A cluster randomized trial	no outcomes
Beeson 2011	Intermittent preventive treatment to reduce the burden of malaria in children: new evidence on integration and delivery	no primary data
Beshir 2016	Baseline frequencies of molecular markers of drug resistance before scaling-up access to seasonal malaria chemoprevention in seven countries across the Sahel	no outcomes
Beshir 2017	Baseline molecular data before scaling-up of seasonal malaria chemoprevention in seven countries across the Sahel	no outcomes
Bicaba 2020	Longitudinal analysis of the capacities of community health workers mobilized for seasonal malaria chemoprevention in Burkina Faso	no outcomes
Bigira 2012	Assessing the association between malaria chemoprevention and the nutritional status of a cohort of young African children	not seasonal
Bigira 2014	Protective efficacy and safety of three antimalarial regimens for the prevention of malaria in young Ugandan children: a randomized controlled trial	not seasonal
Bisanzio 2019	The effectiveness of seasonal malaria chemoprevention (SMC) in the operational programming context of Guinea	no outcomes
Bojang 2008	A randomised trial to compare the safety, tolerability and efficacy of three drug combinations for intermittent preventive treatment in children	duplicate
Bojang 2009	A Study of intermittent preventive treatment and home based management of malaria in a rural area of The Gambia	link to included
Bojang 2010	A randomised trial to compare the safety, tolerability and efficacy of three drug combinations for intermittent preventive treatment in children	no control - compares drug regimens
Bojang 2011	Two strategies for the delivery of IPTc in an area of seasonal malaria transmission in the Gambia: a randomised controlled trial	no control - compares delivery modes
Bonkoungou 2018	Seasonal malaria chemoprevention, an effective intervention for reducing malaria morbidity and mortality	no control
Bonkoungou 2019	Using seasonal malaria chemoprevention (SMC) to screen for acute malnutrition	no outcomes
Boulanger 2010	Immunological consequences of intermittent preventive treatment against malaria in Senegalese preschool children	no outcomes
Brune 2017	Seasonal malaria chemoprevention in Ankililoaka, Madagascar	no control
Cairns 2010	Amodiaquine dosage and tolerability for intermittent preventive treatment to prevent malaria in children	no outcomes

Cairns 2012	Estimating the potential public health impact of seasonal malaria chemoprevention in African children	modeling
Cairns 2015	Analysis of Preventive Interventions for Malaria: Exploring Partial and Complete Protection and Total and Primary Intervention Effects	no control - SMC vs IPTi
Cairns 2015	Randomised controlled trial: Monthly malaria chemoprevention shows potential in an area of very high, perennial malaria transmission	no primary data
Cairns 2016	Optimizing seasonal malaria chemoprevention (SMC) in Africa: Estimating the impact of increasing the number of SMC cycles on the number of children protected, the malaria burden and cost-effectiveness	modeling
Cairns 2017	Monitoring the protective efficacy of seasonal malaria chemoprevention using case-control studies: Methodology and results from 5 countries	case control
Cairns 2019	Effectiveness of seasonal malaria chemoprevention in areas of intense, seasonal malaria transmission: Secondary analysis of data from a household-randomized clinical trial in Burkina Faso and Mali	intervention: SMC+AZ
Cairns 2019	The duration of protection from azithromycin against malaria, pneumonia and gastroenteritis when given alongside seasonal malaria chemoprevention: Secondary analysis of data from a clinical trial in Hounde, Burkina Faso and Bougouni, Mali	intervention: SMC+AZ
Cairns 2020	Evaluation of seasonal malaria chemoprevention in two areas of intense seasonal malaria transmission: Secondary analysis of a household-randomised, placebo-controlled trial in Hounde District, Burkina Faso and Bougouni District, Mali	intervention: SMC+AZ
CairnsMatthew 2015	Monthly malaria chemoprevention shows potential in an area of very high, perennial malaria transmission	duplicate
Camara 2018	Perception of the mothers and the child minders of the region of Sedhiou on the seasonal malaria chemoprevention in 2017: Are the absences and the diseases of the children - No cases of disguised refusals?	no outcomes
Ceesay 2016	Implementation of seasonal malaria chemoprevention in the Gambia	no outcomes
Ceesay 2017	IMPLEMENTATION OF SEASONAL MALARIA CHEMOPREVENTION IN THE GAMBIA	no outcomes
Chandramohan 2019	Effect of Adding Azithromycin to Seasonal Malaria Chemoprevention	intervention: SMC+AZ
Chatio 2019	Community acceptability of Seasonal Malaria Chemoprevention of morbidity and mortality in young children: A qualitative study in the Upper West Region of Ghana	no outcomes
Cisse 2009	Randomized trial of piperaquine with sulfadoxine-pyrimethamine or dihydroartemisinin for malaria intermittent preventive treatment in children	no control - compares drug regimens
Clarke 2008	Effect of intermittent preventive treatment of malaria on health and education in schoolchildren: a cluster-randomised, double-blind, placebo-controlled trial	not seasonal
Clarke 2014	Seasonal malaria chemoprevention and micronutrient supplementation in early childhood: Effect on asymptomatic parasitaemia, anemia and cognition	intervention: SMC+nutrition
Clarke 2015	Seasonal malaria chemoprevention combined with micronutrient supplementation delivered through community preschools: Findings from a cluster randomized trial in Mali	intervention: SMC+nutrition
Clarke 2016	Impact of micronutrient supplementation combined with malaria chemoprevention on malaria, anaemia and	intervention:

	cognitive development in early childhood: Findings from a cluster randomized study in southern Mali	SMC+nutrition
Clarke 2017	Impact of a malaria intervention package in schools on Plasmodium infection, anaemia and cognitive function in schoolchildren in Mali: a pragmatic cluster-randomised trial	intervention: < 3 cycles
Clarke 2017	IMPACT OF MICRONUTRIENT SUPPLEMENTATION COMBINED WITH MALARIA CHEMOPREVENTION ON MALARIA, ANAEMIA AND COGNITIVE DEVELOPMENT IN EARLY CHILDHOOD: FINDINGS FROM A CLUSTER RANDOMIZED STUDY IN SOUTHERN MALIÂ	intervention: SMC+nutrition
Coldiron 2016	Safety and tolerability of dihydroartemisinin-piperaquine as intermittent preventive treatment for malaria in a refugee camp, adjumani, Uganda	not seasonal
Coldiron 2017	PROTECTIVE EFFECTIVENESS OF SEASONAL MALARIA CHEMOPREVENTION IN NIGER: A PROSPECTIVE CASECONTROL STUDY	case control
Coldiron 2017	Prevalence of parasitemia in an area receiving SMC	no control
Coldiron 2017	Prevalence of parasitemia during two seasons in an area receiving seasonal malaria chemoprevention (SMC) in Niger	no control
Coldiron 2017	Intermittent preventive treatment for malaria among children in a refugee camp in Northern Uganda: lessons learned	not seasonal
Coldiron 2019	Clinical diagnostic evaluation of HRP2 and pLDH-based rapid diagnostic tests for malaria in an area receiving seasonal malaria chemoprevention in Niger	no control - comparison of RDTs
Compaore 2011	Efficacy of dihydroartemisinin plus piperaquine compared to amodiaquine plus sulfadoxine/pyrimethamine in seasonal IPT of malaria in children in a rural area of Bobo-Dioulasso (Burkina Faso)	link to included
Compaore 2017	Association of malaria and anemia with malnutrition in children following a seasonal malaria chemoprevention program in a rural area of Burkina Faso	no control
Compaore 2017	Evaluation of the implementation fidelity of the seasonal malaria chemoprevention intervention in Kaya health district, Burkina Faso	no outcomes
Conteh 2010	Cost effectiveness of seasonal intermittent preventive treatment using amodiaquine & artesunate or sulphadoxine-pyrimethamine in Ghanaian children	no outcomes
Danquah 2012	Reduced efficacy of intermittent preventive treatment of malaria in malnourished children (Antimicrobial Agents and Chemotherapy (2009) 53, 5 (1753-1759))	intervention: IPTi
Diallo 2014	Pharmacovigilance during campaign of seasonal malaria chemoprevention in Senegal, 2013	no outcomes
Diallo 2016	Active monitoring of pharmacovigilance at community level during the seasonal malaria chemoprevention campaign in the health district Kolda Senegal, 2015	no control - compares pharmacovigilance
Diallo 2017	Monitoring seasonal malaria chemoprevention campaigns: lessons learned from coverage surveys in 7 countries	no outcomes
Diallo 2018	Monitoring of pharmacovigilance during the seasonal malaria chemoprevention campaign in Senegal, 2013 to 2017	no outcomes
Diarra 2015	Qualitative Research to Inform the Implementation of Home Fortification with Nutrition Education with Seasonal Malaria Chemoprevention through Early Childhood Development Centres for Children Aged 6-59 Months in Sikasso, Mali	no outcomes

Diarra 2017	Impact of micronutrient powders combined with malaria chemoprevention on anemia, malaria and cognitive development: A cluster-randomized study in Malian children	intervention: SMC+nutrition
Diarra 2018	Impact of seasonal malaria chemoprevention on malaria transmission on two sites of therapeutic efficacy study in Mali	no control
Diarra 2019	Molecular studies of PfDHPS and PfDHFR during seasonal malaria chemoprevention at three study sites in Mali	no outcomes
Diawara 2015	Measuring the impact of seasonal malaria chemoprevention as part of routine malaria control in Kita Mali	no control
Diawara 2017	Measuring the impact of seasonal malaria chemoprevention as part of routine malaria control in Kita, Mali	design: one cluster per arm
Diawara 2017	FAILURE OF AVAILABLE MALARIA CONTROL INTERVENTIONS IN DANGASSA, MALI: CONTINUOUSLY HIGH PREVALENCE OF PLASMODIUM FALCIPARUM INFECTION IN A COHORT OF 1,400 INDIVIDUALS FROM 2012 TO 2015	no control
Diawara 2019	Impact of seasonal malaria chemoprevention among children five to ten years of age in Kita and Bafoulabe districts, Mali	abstract only
Dicko 2008	Impact of intermittent preventive treatment with sulphadoxine-pyrimethamine targeting the transmission season on the incidence of clinical malaria in children in Mali	intervention: < 3 cycles
Dicko 2011	Malaria morbidity in children in the year after they had received intermittent preventive treatment of malaria in Mali: a randomized control trial	link to included
Dicko 2016	A trial of seasonal malaria chemoprevention plus azithromycin in African children	protocol
Dieng 2019	Contrasting Asymptomatic and Drug Resistance Gene Prevalence of Plasmodium falciparum in Ghana: Implications on Seasonal Malaria Chemoprevention	no outcomes
Ding 2020	Adherence and Population Pharmacokinetic Properties of Amodiaquine When Used for Seasonal Malaria Chemoprevention in African Children	no outcomes
Diouf 2015	Seasonal malaria chemoprevention implementation in children from three to 120 months experience in the four southern regions in Senegal	no outcomes
Druetz 2017	Seasonal malaria chemoprevention in Burkina Faso protects children against malaria and anaemia under routine program implementation	inappropriate control
Druetz 2018	Impact Evaluation of Seasonal Malaria Chemoprevention under Routine Program Implementation: A Quasi-Experimental Study in Burkina Faso	inappropriate control
Druetz 2018	Evaluation of direct and indirect effects of seasonal malaria chemoprevention in Mali	no control
Esch 2018	Estimating the health impact of a seasonal malaria chemoprevention intervention in Mali in 2017: Modeling deaths averted, cases averted and disability adjusted life years (DALYS) averted	modeling
Esch 2019	Prospectively estimating the health impact of upcoming president's malaria initiative impact malaria project-supported seasonal malaria chemoprevention campaigns in 70 districts across Niger, Mali and Cameroon in 2019	modeling
Fabrice 2010	Prevalence and selection of Plasmodium falciparum drug resistance molecular markers under intermittent preventive therapy in Burkina Faso	no outcomes
Fabrice 2011	Plasmodium falciparum drug resistance molecular markers under intermittent preventive therapy with	no outcomes

	dihydroartemisinin-piperaquine (DP) vs. amodiaquine-sulfadoxine/pyrimethamine (AQ-SP) in Burkina Faso	
Geerligs 2003	Analysis of the effects of malaria chemoprophylaxis in children on haematological responses, morbidity and mortality	review
Grais 2018	Molecular markers of resistance to amodiaquine plus sulfadoxine-pyrimethamine in an area with seasonal malaria chemoprevention in south central Niger	no outcomes
Gueye 2018	Decrease of malaria burden among children under five years and other age groups in SMC regions in Senegal	no control
Guillebaud 2013	Epidemiology of malaria in an area of seasonal transmission in Niger and implications for the design of a seasonal malaria chemoprevention strategy	no outcomes
Hulle 2016	Implementing seasonal malaria chemoprevention (SMC) in the context of Ebola virus disease (EVD) in Guinea	no control
Humphreys 2018	Spatiotemporal modelling of prevalence of plasmodium falciparum drug resistance mutations in the DHPS gene across Africa, 1990 - 2015	modeling
Issiaka 2015	Determining the optimal mode for delivery of seasonal malaria chemoprevention in Mali	no outcomes
Issiaka 2017	Impact of seasonal malaria chemoprevention on hospital admissions and mortality in children under 5 years in Ouelessebougou, Mali	link to included
Jagannathan 2016	Effective Antimalarial Chemoprevention in Childhood Enhances the Quality of CD4+ T Cells and Limits Their Production of Immunoregulatory Interleukin 10	no outcomes
Jean 2012	Seasonal malaria chemoprevention and community case management for malaria in southern Senegal: A cluster-randomized trial	no outcomes
Kaly 2018	Knowledge, attitudes and practices of mothers caretakers of children aged 3 to 120 months on of seasonal malaria chemo prevention in Bounkiling health district, South Senegal	no outcomes
Kamate 2017	Scaling up seasonal malaria chemoprevention in Mali: Implementation challenges and lessons learned	no control
Kamya 2020	The Impact of Control Interventions on Malaria Burden in Young Children in a Historically High-Transmission District of Uganda: A Pooled Analysis of Cohort Studies from 2007 to 2018	not seasonal
Kana 2017	Scaling-up of seasonal malaria chemoprevention in Sokoto and Zamfara states, Nigeria: Monitoring delivery and impact	no outcomes
Katile 2019	Malaria parasitemia incidence among different age groups in a stable transmission area of Mali receiving seasonal malaria chemoprevention	no control
Kobbe 2011	Follow-up survey of children who received sulfadoxine-pyrimethamine for intermittent preventive antimalarial treatment in infants	intervention: IPTi
Kombate 2019	Analysis of the quality of seasonal malaria chemoprevention provided by community health Workers in Boulsa health district, Burkina Faso	no outcomes
Konate 2011	Morbidity from malaria in children in the year after they had received intermittent preventive treatment of malaria: a randomised trial	link to included
Konate 2019	Effect of seasonal malaria chemoprevention on malaria in children under 5 years: A cohort study in Dangassa, Mali	no control
Konate 2020	Effect of routine seasonal malaria chemoprevention on malaria trends in children under 5 years in Dangassa,	no control

	Mali	
Koscalova 2015	Monitoring the protective effect and the effectiveness of seasonal malaria chemoprevention in Niger	inappropriate control
Koscalova 2017	Malaria incidence in the area of seasonal malaria chemoprevention	case control
Kpormegbe 2014	The role of community participation in intermittent preventive treatment of childhood malaria in southeastern Ghana	no outcomes
Kweku 2009	Options for the delivery of intermittent preventive treatment for malaria to children: a community randomised trial	no outcomes
Kweku 2011	Assessment of the efficacy, tolerability and ease of administration of dihydroarthemisinin plus piperaquine and artesunate plus sulfamethoxypyrazine plus pyrimethamine compared with sulphadoxine-pyrimethamine for preventing malaria in Ghanaian children	no control
Lasry 2015	Seasonal malaria chemoprevention, three years of implementation	no control
LeMenach 2015	Combining nutritional supplementation with seasonal malaria chemoprevention in Nigeria decreases the odds of confirmed clinical malaria: Findings from a case-control study	intervention: SMC+nutrition
Lo 2011	Impact of seasonal intermittent preventive treatment in children: Molecular markers of resistance in three health districts in Senegal	no outcomes
Lo 2012	Prevalence of mutation of pfcrt after the use of amodiaquine in intermittent preventive treatment in children (IPTC) in Senegal	no outcomes
Lo 2013	Prevalence of molecular markers of drug resistance in an area of seasonal malaria chemoprevention in children in Senegal	no outcomes
Mahamar 2016	Seasonal malaria chemoprevention is associated with a reduction in seropositivity to blood-stage antigens	no outcomes
Mahamar 2017	Effect of seasonal malaria chemoprevention on the acquisition of antibodies to Plasmodium falciparum antigens in Ouelessebougou, Mali	no outcomes
Mahamar 2019	Long-term effect of seasonal malaria chemoprevention with amodiaquine plus sulfadoxine-pyrimethamine on molecular markers of resistance in Ouelessebougou, Mali	no outcomes
Mahamar 2019	Effect of four years of seasonal malaria chemoprevention on the acquisition of antibodies to plasmodium falciparum antigens in Ouelessebougou, Mali	no outcomes
Maiga 2014	School performance after intermittent preventive treatment using artemisinin-based combination	intervention: < 3 cycles
Maiga 2016	Seasonal Malaria Chemoprevention with Sulphadoxine-Pyrimethamine and Amodiaquine Selects Pfdhfr-dhps Quintuple Mutant Genotype in Mali	no outcomes
Maiga 2018	Selection of seven-mutation Pfcrt-Pfmdr1 genotype after scaling seasonal malaria chemoprevention with sulphadoxine-pyrimethamine and amodiaquine in Mali	no outcomes
Maiteki 2015	School-based treatment with act to reduce transmission' (start-IPT): Evaluation of the community impact of intermittent preventive treatment for malaria in Ugandan children	not seasonal
Maiteki-Sebuguzi 2017	EVALUATING THE COMMUNITY-LEVEL IMPACT OF INTERMITTENT PREVENTIVE TREATMENT OF SCHOOLCHILDREN FOR MALARIA IN JINJA, UGANDA: A CLUSTER-RANDOMIZED TRIAL	not seasonal

Matangila 2015	Efficacy and safety of intermittent preventive treatment with sulfadoxine-pyrimethamine (SP) and SP-piperaquine in schoolchildren in Kinshasa, The Democratic Republic of the Congo (RDC)	not seasonal
Matangila 2015	Efficacy and safety of intermittent preventive treatment for malaria in schoolchildren: a systematic review	review
Matangila 2017	The perception of parents and teachers about intermittent preventive treatment for malaria in school children in a semi-rural area of Kinshasa, in the Democratic Republic of Congo	not seasonal
Matangila 2017	Efficacy and safety of intermittent preventive treatment in schoolchildren with sulfadoxine/pyrimethamine (SP) and SP plus piperaquine in Democratic Republic of the Congo: a randomised controlled trial	not seasonal
Meremikwu 2005	Chemoprophylaxis and intermittent treatment for preventing malaria in children	review
Meremikwu 2008	Chemoprophylaxis and intermittent treatment for preventing malaria in children	review
Meremikwu 2012	Intermittent preventive treatment for malaria in children living in areas with seasonal transmission	review
Moroso 2017	Transforming the malaria landscape: Results from a three year implementation research project expanding access to seasonal malaria chemoprevention in seven Sahelian countries (ACCESS-SMC)	case control
Muhindo 2017	Intermittent preventive treatment with dihydroartemisinin-piperaquine in young Ugandan children in the setting of indoor residual spraying of insecticide	not seasonal
Namirimu 2013	Impact of chemoprevention on the development of t cell responses to malaria	not seasonal
Nankabirwa 2010	Efficacy, safety, and tolerability of three regimens for prevention of malaria: a randomized, placebo-controlled trial in Ugandan schoolchildren	not seasonal
Nankabirwa 2014	Impact of intermittent preventive treatment with dihydroartemisinin-piperaquine on malaria in Ugandan schoolchildren: a randomized, placebo-controlled trial	not seasonal
National Institute for Medical Research 2020	Evaluation of the Implementation and Effectiveness of Intermittent Preventive Treatment for Malaria Using Dihydroartemisinin-piperaquine on Reducing Malaria Burden in School Aged Children in Tanzania	not seasonal
Nayiga 2015	Lessons for integrating intermittent preventive treatment for malaria in school systems in low income settings: Experiences from Uganda	not seasonal
Nct 2005	Study of the Impact of Intermittent Preventive Treatment in Schools on Malaria, Anaemia and Education	protocol
Nct 2014	A Trial of Seasonal Malaria Chemoprevention Plus Azithromycin in African Children	protocol
Nct 2017	Seasonal Malaria Chemoprevention With or Without Lipid-based Nutrient Supplement in Children Aged 6-59 Months in Mali	protocol
Nct 2019	Seasonal Malaria Chemoprevention With Dihydroartemisin Piperaquine vs. Sulfadoxine-pyrimethamine+Amodiaquine	protocol
Ndiaye 2011	Costing a large-scale implementation of intermittent preventive treatment of malaria in children delivered through community health workers in Senegal	no outcomes

Ndiaye 2011	Impact of intermittent preventive treatment in children (IPTC) on Plasmodium falciparum infections complexity: Resistance markers and kinetic of antibodies against P. falciparum in Senegal	no outcomes
Ndiaye 2011	Monitoring of drug resistance after intermittent preventive treatment for infants and children (IPTI/C) in Senegal	no outcomes
Ndiaye 2011	Safety of seasonal intermittent preventive treatment against malaria with sulfadoxine pyrimethamine + amodiaquine when delivered to children under ten years of age by district health staff in Senegal	no outcomes
Ndiaye 2012	Costing a large-scale implementation of seasonal malaria chemoprevention in children delivered through community health workers in Senegal	no outcomes
Ndiaye 2013	Evaluation of the tolerance of sulfadoxine-pyrimethamine + amodiaquine combination in seasonal malaria chemoprevention (SMC) combined with home based management (HMM) in children under 10 years in Senegal	link to included
Ndiaye 2013	Selection of antimalarial drug resistance after intermittent preventive treatment of infants and children (IPTi/c) in Senegal	no outcomes
Ndiaye 2013	Seasonal malaria chemoprevention in Senegal: From research to policy	no outcomes
Ndiaye 2014	Potential impact of intermittent preventive treatment (IPT) on the acquisition of antibodies to malaria antigens GLURP-R0 and AMA-1 in Senegalese children	no outcomes
Ndiaye 2015	Evaluation of the impact of seasonal malaria chemoprevention administered by mass campaign in Southern Senegal	case control
Ndiaye 2015	Potential Impact of Seasonal Malaria Chemoprevention on the Acquisition of Antibodies Against Glutamate-Rich Protein and Apical Membrane Antigen 1 in Children Living in Southern Senegal	no outcomes
Ndiaye 2016	Impact evaluation after three years of seasonal malaria chemoprevention implementation by mass campaigns in southern Senegal	case control
Ndiaye 2016	Safety of Seasonal Malaria Chemoprevention (SMC) with Sulfadoxine-Pyrimethamine plus Amodiaquine when Delivered to Children under 10 Years of Age by District Health Services in Senegal: Results from a Stepped-Wedge Cluster Randomized Trial	link to included
Ndiaye 2016	Trends of high reduction of malaria cases in Sedhiou district following seasonal malaria chemoprevention first campaign: Lessons learned	no control
Ndiaye 2016	IMMUNOLOGICAL EFFECT OF SEASONAL MALARIA CHEMOPREVENTION (SMC) WITH SULFADOXINEPYRIMETHAMINE (SP) AND AMODIAQUINE (AQ) IN CHILDREN UNDER 10 YEARS IN THE SOUTHEASTERN PART OF SENEGAL	no outcomes
Ndiaye 2017	IMPACT EVALUATION AFTER THREE YEARS OF SEASONAL MALARIA CHEMOPREVENTION IMPLEMENTATION BY MASS CAMPAIGNS IN SOUTHERN SENEGAL	case control
Ndiaye 2017	TRENDS OF HIGH REDUCTION OF MALARIA CASES IN SEDHIOU DISTRICT FOLLOWING SEASONAL MALARIA CHEMOPREVENTION FIRST CAMPAIGN: LESSONS LEARNEDÂ	no control
Ndiaye 2017	Impact of seasonal malaria chemoprevention after 3 years at scale in Southern Senegal	no control
Ndiaye 2018	Evaluation of Two Strategies for Community-Based Safety Monitoring during Seasonal Malaria Chemoprevention Campaigns in Senegal, Compared with the National Spontaneous Reporting System	no outcomes
Ndiop 2015	Tracking the impact of seasonal malaria chemoprevention on morbidity and mortality of children in Senegal	no control

	through the routine health information system	
Ndiop 2017	Has seasonal malaria chemoprevention decreased the malaria burden among children under five years in Senegal?	no control
Nonvignon 2016	Cost-effectiveness of seasonal malaria chemoprevention in upper west region of Ghana	no outcomes
Noor 2015	Sub-National Targeting of Seasonal Malaria Chemoprevention in the Sahelian Countries of the Nouakchott Initiative	modeling
Ntab 2007	Impact of intermittent preventive anti-malarial treatment on the growth and nutritional status of preschool children in rural Senegal (west Africa)	no outcomes
Obiero 2019	The effect of adding azithromycin to the antimalarials (sulphadoxine/pyrimethamine and amodiaquine) used for seasonal malaria chemoprevention on the immune response to plasmodium falciparum	no outcomes
Okuneye 2019	Using models to inform implementation policies of seasonal malaria chemoprevention	modeling
Oresanya 2019	An assessment of quality of delivery of seasonal malaria chemoprevention using low literate community health workers in Nigeria	no outcomes
Ouedraogo 2016	Impact of seasonal malaria chemoprophylaxis in a high and seasonal malaria transmission setting in Burkina Faso	modeling
Ouedraogo 2016	Effective scaling-up of seasonal malaria chemoprevention in Burkina Faso	no outcomes
Ouedraogo 2017	Understanding and optimizing operational seasonal malaria chemoprevention through data analysis and modeling: The example of Burkina Faso	modeling
Ouedraogo 2017	EFFECTIVE SCALING-UP OF SEASONAL MALARIA CHEMOPREVENTION IN BURKINA FASO	no outcomes
Pactr 2013	An integrated malaria control strategy including community case management and Seasonal Malaria Chemoprevention in Senegal	protocol
Pactr 2014	Randomized open-label trial to evaluate the efficacy of artesunate-amodiaquine for seasonal malaria chemoprevention in suburban school of Bamako, Sirak	protocol
Patouillard 2011	Coverage, adherence and costs of intermittent preventive treatment of malaria in children employing different delivery strategies in Jasikan, Ghana	no outcomes
Pitt 2012	Intermittent preventive treatment of malaria in children: a qualitative study of community perceptions and recommendations in Burkina Faso and Mali	no outcomes
Pitt 2015	Delivering seasonal malaria chemoprevention to children under ten at scale in central Senegal: Costs and cost determinants	no outcomes
Pitt 2017	Large-scale delivery of seasonal malaria chemoprevention to children under 10 in Senegal: an economic analysis	no outcomes
Rehman 2019	Intermittent preventive treatment of malaria delivered to primary schoolchildren provided effective individual protection in Jinja, Uganda: secondary outcomes of a cluster-randomized trial (START-IPT)	not seasonal
Roger 2011	Impact of combining intermittent preventive treatment with home management of malaria in children under ten	intervention: < 3

	years, in a rural area of Senegal	cycles
Rohner 2010	In a randomized controlled trial of iron fortification, anthelmintic treatment, and intermittent preventive treatment of malaria for anemia control in Ivorian children, only anthelmintic treatment shows modest benefit	intervention: < 3 cycles
Sagara 2016	Coverage of seasonal malaria chemoprevention delivered by mobile teams at fixed points in 14 districts in Mali, through Access-SMC	no outcomes
Sagara 2017	A TRIAL OF SEASONAL MALARIA CHEMOPREVENTION PLUS AZITHROMYCIN IN AFRICAN CHILDREN	intervention: SMC+AZ
Sagara 2017	Seasonal malaria chemoprevention scaling up and its impact assessment in Mali	no control
Sagara 2017	COVERAGE OF SEASONAL MALARIA CHEMOPREVENTION DELIVERED BY MOBILE TEAMS AT FIXED POINTS IN 14 DISTRICTS IN MALI, THROUGH ACCESS-SMC	no outcomes
Sagara 2018	Seasonal malaria chemoprevention scaling up in Mali: Coverage and impact on malaria burden and markers of the resistance of plasmodium falciparum to sulfadoxine pyrimethamine and amodiaquine	no control
Salam 2014	Impact of community-based interventions for the prevention and control of malaria on intervention coverage and health outcomes for the prevention and control of malaria	review
Salissou 2016	Perception de la chimioprevention du paludisme saisonnier au Niger	duplicate
Salissou 2016	Perception of the seasonal malaria chemoprevention in Niger	no outcomes
Sangare 2019	Seasonal malaria chemoprevention and compliance during four monthly treatments with sulfadoxine-pyrimethamine and amodiaquine at 3 study sites in Mali	no outcomes
Saye 2015	Malaria chemoprevention, undernutrition and anaemia in children: Findings from three randomized intervention trials in Southern Mali	intervention: SMC+nutrition
Shehu 2017	Malaria preventive practices and acceptability of seasonal malaria chemoprevention among caregivers of under five children in rural and urban communities of Kano, Nigeria, 2017	no outcomes
Sokhna 2008	A trial of the efficacy, safety and impact on drug resistance of four drug regimens for seasonal intermittent preventive treatment for malaria in Senegalese children	no control - comparison of 4 regimens
Some 2014	Selection of drug resistance-mediating Plasmodium falciparum genetic polymorphisms by seasonal malaria chemoprevention in Burkina Faso	no outcomes
Soumare 2017	SEASONAL MALARIA CHEMOPREVENTION IS ASSOCIATED WITH A REDUCTION IN SEROPOSITIVITY TO BLOOD-STAGE ANTIGENS	no outcomes
Staedke 2018	Assessment of community-level effects of intermittent preventive treatment for malaria in schoolchildren in Jinja, Uganda (START-IPT trial): a cluster-randomised trial	not seasonal
Strachan 2016	The use of formative research to inform the design of a seasonal malaria chemoprevention intervention in northern Nigeria	no outcomes
Sundell 2015	Variable piperaquine exposure significantly impacts protective efficacy of monthly dihydroartemisinin-piperaquine for the prevention of malaria in Ugandan children	not seasonal
Sylla 2016	Immunological effect of seasonal malaria chemoprevention (SMC) with sulfadoxine-pyrimethamine (SP) and	no outcomes

	amodiaquine (AQ) in children under 10 years in the southeastern part of Senegal	
Tagbor 2015	The protective efficacy of seasonal malaria chemoprevention in an area of extended seasonal transmission in the Ashanti region of Ghana: An individually randomized trial	link to included
Tarning 2018	Pharmacokinetic and pharmacodynamic properties of dihydroartemisinin-piperaquine in seasonal malaria chemoprevention in young children	no outcomes
Temperley 2008	Costs and cost-effectiveness of delivering intermittent preventive treatment for malaria through schools in western Kenya	not seasonal
Temperley 2008	Costs and cost-effectiveness of delivering intermittent preventive treatment through schools in western Kenya	not seasonal
Thomas 2017	Malaria prevention with nutrient supplementation in addition to seasonal chemoprevention in children aged 6-59 months in rural Mali	intervention: SMC+nutrition
Tine 2011	Impact of combining intermittent preventive treatment with home management of malaria in children under 10 years, in a rural area of Senegal	intervention: < 3 cycles
Tine 2011	Impact of combining intermittent preventive treatment with home management of malaria in children less than 10 years in a rural area of Senegal: a cluster randomized trial	intervention: < 3 cycles
Tine 2013	Feasibility, safety and effectiveness of combining home based malaria management (HMM) and seasonal malaria chemoprevention (SMC) in children less than ten years in Senegal: A cluster-randomized trial	link to included
Tine 2013	Acceptability by community health workers in Senegal of combining community case management of malaria and seasonal malaria chemoprevention	no outcomes
Tine 2014	Combining community case management of malarial and seasonal malaria chemoprevention for children less than 10 years in Senegal: Feasibility, impact on malaria and Anemia	link to included
Toure 2016	Failure of available malaria control interventions in Dangassa, Mali: Continuously high prevalence of plasmodium falciparum infection in a cohort of 1,400 individuals from 2012 to 2015	no control
Toure 2017	Age-specific changes in the incidence of uncomplicated plasmodium falciparum malaria: Seasonal malaria chemoprevention (SMC) in an area with intense transmission: Dangassa, Mali	abstract only
VanHulle 2017	IMPLEMENTING SEASONAL MALARIA CHEMOPREVENTION (SMC) IN THE CONTEXT OF EBOLA VIRUS DISEASE (EVD) IN GUINEA	no control
Wagman 2017	Observational evidence of a complimentary effect of combining next generation indoor residual spraying and seasonal malaria chemoprevention in the Segou region of Mali, 2014	link to included
Ward 2014	Comparison of seasonal malaria chemoprevention coverage in northern Nigeria via door-to-door, health facility and retail sector delivery	no outcomes
Ward 2015	Impact of integrating the delivery of seasonal malaria chemoprevention with nutrition supplementation in northern Nigeria on health outcomes: A pragmatic intervention trial	intervention: SMC+nutrition
Ward 2019	Seasonal malaria chemoprevention packaged with malnutrition prevention in northern Nigeria: A pragmatic trial (SMAMP study) with nested case-control	intervention: SMC+nutrition
Wilson 2011	A systematic review and meta-analysis of the efficacy and safety of intermittent preventive treatment of malaria	review

	in children (IPTc)	
Yerbanga 2016	Assessment of malaria transmission from human to mosquitoes in seasonal malaria chemoprevention in the western region of Burkina Faso	no outcomes
York 2017	Seasonal malaria chemoprevention in the Sahel	no primary data
Zongo 2016	Dihydroartemisin-piperaquine for seasonal malaria chemoprevention	no control
Zongo 2017	DIHYDROARTEMISIN-PIPERAQUINE FOR SEASONAL MALARIA CHEMOPREVENTION	no control
Zongo 2019	Optimizing delivery of seasonal malaria chemoprevention (SMC) for children under five years of age: Very high coverage consistently achieved through door-to-door campaigns in Burkina Faso	no outcomes
Zuilkowski 2014	Early childhood malaria prevention and children's patterns of school leaving in the Gambia	no outcomes

AZ: azithromycin; IPTi: intermittent preventive treatment in infancy; SMC: seasonal malaria chemoprevention; RDT: rapid diagnostic test

	Chemoprevention OR chemoprophylaxis OR Chemo-prevention OR chemo-prophylaxis OR “intermittent preventive”<br>AND<br>Child* OR school* OR preschool* OR infant* OR newborn* OR neonat*<br>Limit 2000 – ;		duplicates<br>=9 unique items
**CINAHL (EBSCOHost)**	Malaria* OR antimalaria* OR anti-malaria*<br>AND<br>Chemoprevention OR chemoprophylaxis OR Chemo-prevention OR chemo-prophylaxis OR “intermittent preventive”<br>AND<br>Child* OR school* OR preschool* OR infant* OR newborn* OR neonat*<br>Limit 2000 – ; Exclude Medline records	3/2/2021	3<br>-2 duplicates<br>=1 unique items
**Africa-Wide Information (EBSCOHost)**	Malaria* OR antimalaria* OR anti-malaria*<br>AND<br>Chemoprevention OR chemoprophylaxis OR Chemo-prevention OR chemo-prophylaxis OR “intermittent preventive”<br>AND<br>Child* OR school* OR preschool* OR infant* OR newborn* OR neonat*<br>Limit 2000 – ;	3/2/2021	57<br>-55 duplicates<br>=2 unique items
**Scopus**	TITLE-ABS-KEY(Malaria* OR antimalaria* OR anti-malaria*) AND TITLE-ABS-KEY(Chemoprevention OR chemoprophylaxis OR Chemo-prevention OR chemo-prophylaxis OR “intermittent preventive”) AND TITLE-ABS-KEY(Child* OR school* OR preschool* OR infant* OR newborn* OR neonat*) AND NOT INDEX(medline)<br>Limit 2000 – ;	3/2/2021	47<br>-41 duplicates<br>=6 unique items
**Global Index Medicus (WHO)**<br>**-includes LILACS**	Malaria* OR antimalaria* OR anti-malaria*<br>AND<br>Chemoprevention OR chemoprophylaxis OR Chemo-prevention OR chemo-prophylaxis OR “intermittent preventive”<br>AND<br>Child* OR school* OR preschool* OR infant* OR newborn* OR neonat*<br>Limit 2000 – ;	3/2/2021	24<br>-5 duplicates<br>=19 unique items
**Clinicaltrails.gov**	intermittent preventive treatment OR intermittent preventive therapy | malaria | Child (birth – 17)	3/2/2021	62<br>-0 duplicates<br>=62 unique items

Notes: Duplicates were identified using the Endnote automated "find duplicates" function with preference set to match on title, author and year, and removed from your Endnote library. There will likely be additional duplicates found that Endnote was unable to detect.

Supplemental File 1: Non-randomized study designs

Types of studies included:

- Controlled before-and-after studies (CBAs) with: (a) a contemporaneous control group, and (b) at least two sites per arm.
- Interrupted time series (ITS) studies with: (a) a clearly defined point in time when the intervention occurred, and (b) at least two (if a contemporaneous control group is available) or three (if no control group is available) data points collected both before the first round of SMC and after the last round of SMC, measured at evenly spaced intervals (i.e., monthly). Baseline up to one year prior to intervention is required.

Description of the search strategy and data abstraction methods are detailed in the main text.

Of the five non-randomized designs included, one was an individual non-randomized controlled trial, which compared SP+AQ and dihydroartemisinin-piperaquine (DP) in a randomized fashion, but did not include a randomized control group (Zongo 2015, Burkina Faso). Four were cluster non-randomized controlled: one used artemether-lumefantrine (AL) in three cycles among school children with anti-parasitics (albendazole+praziquantel) in both arms (Opoku 2016, Ghana), two compared four cycles of SP+AQ vs nothing among children <5 years of age (Issiaka 2020 in Mali and Salissou 2017 in Niger), and one compared indoor residual spraying (IRS)+SMC vs IRS vs SMC vs nothing in Mali, in which SMC was given as four cycles of SP+AQ to children < 5 years (Wagman 2020). For Zongo 2015, we presented the DP vs control and SP+AQ vs control separately, and for Wagman 2020, we presented SMC+IRS vs IRS and SMC vs nothing separately.

Table SF1.1: Outcomes presented by each study – non-randomized designs

Study	Country	Design	Age group	# of cycles	Comparison	Outcomes
Issiaka 2020	Mali	cluster non-randomized controlled	3-59 months	4	SP+AQ vs nothing	hospitalization, severe malaria, mortality
Opoku 2016	Ghana	cluster non-randomized controlled	6-15 years	3	AL+anti-parasitic vs anti-parasitic	prevalence, anemia
Salissou 2017	Niger	cluster non-randomized controlled	3-59 months	4	SP+AQ vs nothing	incidence, severe malaria, hospitalization, mortality, prevalence, anemia
Wagman 2020	Mali	cluster non-randomized controlled	3-59 months	4	SP+AQ + IRS vs IRS vs SP+AQ vs nothing	incidence
Zongo 2015	Burkina Faso	individual non-randomized control	3-59 months	3	SP+AQ vs control, DP vs control	incidence, mortality, prevalence, anemia

SP: sulfadoxine-pyrimethamine; AQ: amodiaquine; IRS: indoor residual spraying; DP: dihydroartemisinin-piperaquine

Table SF1.2. Characteristics of included non-randomized studies

Study	Country	Design	Age group	Comparison	# of cycles	Number enrolled	SMC coverage	Parasite prevalence	ITN use
Issiaka 2020	Mali	Cross sectional survey	3-59 mo	SP+AQ vs nothing monthly	4	Survey:<br>SMC 2759<br>control 3879	Not reported	50%	>80
Opoku 2016	Ghana	Cluster controlled (2 clusters per arm)	6-15 years	AL+alb+praz vs alb+praz	3	AL+alb+praz: 90<br>alb+praz:127	Not reported	Not reported	40
Salissou 2017	Niger	Cross sectional survey, 48 villages	3-59 mo	SP+AQ monthly vs nothing	4	Survey:<br>241 SMC<br>241 control	Not reported	56%	
Wagman 2020	Mali	Cluster controlled	3-59 mo	SP+AQ monthly + IRS vs IRS vs SP+AQ monthly vs nothing	4	Pop:<br>IRS+SMC: 334,000<br>IRS: 236,000<br>SMC 394,000<br>control 1.42M	>90% (adminis-trative coverage)	50%	>80
Zongo 2015	Burkina Faso	Individual controlled	3-59 mo	SP+AQ vs control, DP vs control, monthly	3	DP: 757<br>SP+AQ: 742<br>Control: 250	>95% per cycle	40-60%	

SP: sulfadoxine-pyrimethamine; AQ: amodiaquine; IRS: indoor residual spraying; DP: dihydroartemisinin-piperaquine; alb: albendazole; praz: praziquantel

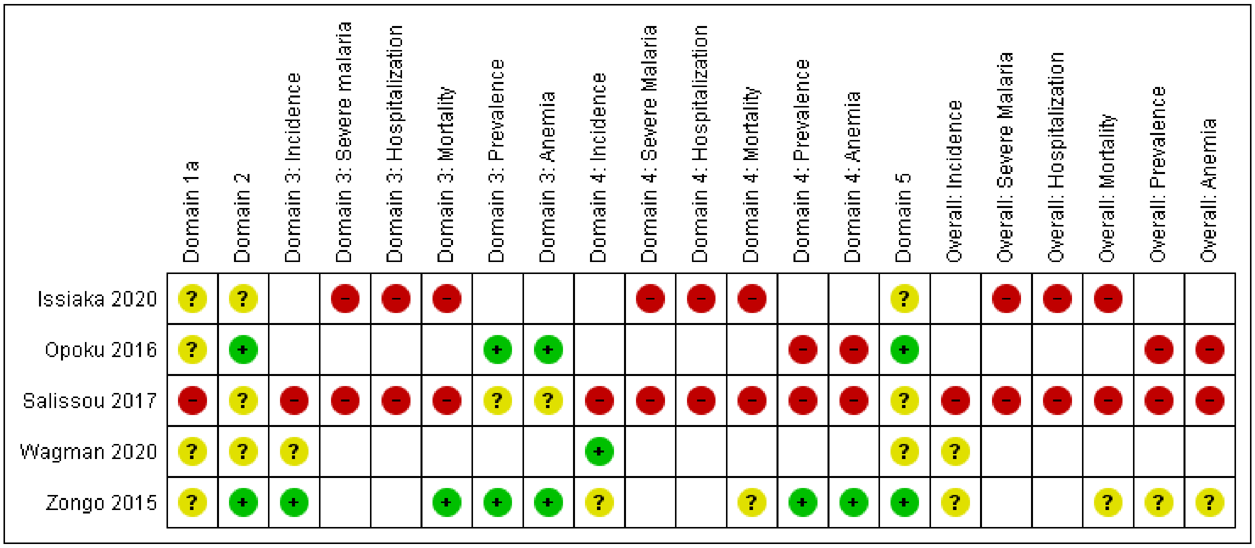

Non-randomized studies: Domain 1a. Failure to develop and apply appropriate eligibility criteria (inclusion of control population); Domain 1b. Not applicable; Domain 2. Flawed measurement of exposure; Domain 3. Incomplete follow-up; Domain 4. Flawed measurement of outcome (outcome-level); Domain 5. Failure to adequately control for confounding

Figure SF1.1. Risk of bias summary: review authors' judgements about each risk-of-bias item for each included study by malaria outcomes, non-randomized studies

Incidence in non-randomized studies

Three studies with non-randomized controls, two of which had multiple controls, reported effect on incidence, all among children < 5 years of age receiving three to four cycles. Zongo 2015 compared SP+AQ to DP in a randomized fashion, but the control group was not randomized. SP+AQ and DP had an effect that was comparable within the trial, and with randomized studies among children < 5 years receiving three to four cycles.

Table SF1.3. Incidence among children < 5 years in non-randomized studies

Study IDs	Country	Comparator	Age group	# of cycles	N	Risk ratio
Salissou 2017	Niger	SP+AQ vs nothing	3-59 mo	4	241 SMC, 241 control	PR: 0.25 (0.20-0.31)
Wagman 2020A	Mali	SP+AQ + IRS vs IRS	3-59 mo	4	IRS+SMC: 334,000; IRS: 236,000,	RR: 0.96 (95% CI 0.51-1.37)
Wagman 2020B	Mali	SP+AQ vs nothing	3-59 mo	4	SMC 394,000; control 1.42M	RR: 0.84 (95% CI 0.45-1.55)
Zongo 2015A	Burkina Faso	SP+AQ vs nothing	3-59 mo	3	742 SP+AQ, 250 control	RR : 0.20 (95% CI 0.14-0.28)
Zongo 2015B	Burkina Faso	DP vs nothing	3-59 mo	3	757 DP, 250 control	RR: 0.26 (95% CI 0.19-0.35)

SP: sulfadoxine-pyrimethamine; AQ: amodiaquine; IRS: indoor residual spraying; DP: dihydroartemisinin-piperaquine; SMC: seasonal malaria chemoprevention

Prevalence among children < 5 years of age in non-randomized designs

Three studies reported prevalence among children < 5 years of age, one that compared SP+AQ to DP (Zongo 2015), one that compared AL + anti-parasitics vs anti-parasitics (Opoku 2016), and one that compared SP+AQ to nothing (Salissou 2017).

Table SF1.4. Prevalence among children < 5 years of age in non-randomized studies

Study ID	Country	Comparator	Age group	# of cycles	N	Prevalence ratio
Opoku 2016	Ghana	AL alb+praz vs alb+praz	6-15 years	3	AL+alb+praz: 90 alb+praz:127	PR: 0.46 (95% CI 0.21-0.98)
Salissou 2017	Niger	SP+AQ vs nothing	3-59 mo	4	241 SMC, 241 control	PR: 0.76 (95% CI 0.57-1.02)
Zongo 2015A	Burkina Faso	SP+AQ vs nothing	3-59 mo	3	742 SP+AQ, 250 control	PR: 0.42 (95% CI 0.31-0.55)
Zongo 2015B	Burkina Faso	DP vs nothing	3-59 mo	3	757 DP, 250 control	PR: 0.49 (95% CI 0.37-0.65)

SP: sulfadoxine-pyrimethamine; AQ: amodiaquine ; AL: artemether-lumefantrine; alb: albendazole; praz: praziquantel; DP: dihydroartemisinin-piperaquine; SMC: seasonal malaria chemoprevention

Prevalence of any anemia among children < 5 years of age in non-randomized studies

Two studies reported any anemia: Opoku 2016 compared AL + albendazole + praziquantel to albendazole + praziquantel in school children 6–15 years of age and Salissou 2017 compared SP+AQ to nothing among children < 5 years.

Table SF1.5. Prevalence of any anemia among children < 5 years, non-randomized studies

Study IDs	Country	Comparator	Age group	# of cycles	N	Prevalence ratio
Opoku 2016	Ghana	AL+alb+praz vs alb+praz	6-15 years	3	AL+alb+praz: 90 alb+praz:127	PR: 1.24 (95% CI 0.62-2.50)
Salissou 2017	Niger	SP+AQ vs nothing	3-59 mo	4	241 SMC, 241 control	PR: 1.0 (95% CI 0.25-3.95)

SP: sulfadoxine-pyrimethamine; AQ: amodiaquine; AL: artemether-lumefantrine; alb: albendazole; praz: praziquantel; SMC: seasonal malaria chemoprevention

Prevalence of moderate anemia among children < 5 years of age in non-randomized studies

Zongo 2015 reported moderate anemia among children < 5 years of age who received either three cycles of SP+AQ or DP to nothing. There was not an effect of SMC, either with SP+AQ or DP, in either arm.

Table SF1.6. Prevalence of moderate anemia among children < 5 years in non-randomized studies

Study IDs	Country	Comparator	Age group	# of cycles	N	Prevalence ratio
Zongo 2015A	Burkina Faso	SP+AQ vs nothing	3-59 mo	3	742 SP+AQ, 250 control	PR = 1.14 (95% CI = 0.80-1.61)
Zongo 2015B	Burkina Faso	DP vs nothing	3-59 mo	3	757 DP, 250 control	PR = 1.04 (95% CI 0.73-1.48)

SP: sulfadoxine-pyrimethamine; AQ: amodiaquine; DP: dihydroartemisinin-piperaquine

Incidence of severe malaria in non-randomized studies

Two studies reported incidence of severe malaria among children < 5 years of age receiving three to four cycles of SP+AQ (Issiaka 2020, Salissou), both based on reports by guardians of children during cross sectional surveys. Results from both studies were comparable to those found in randomized studies.

Table SF1.7. Incidence of severe malaria among children < 5 years, non-randomized studies

Study ID	Country	Comparator	Age group	# of cycles	N	Risk ratio
Issiaka 2020	Mali	SP+AQ vs nothing	3-59 mo	4	2759 SMC, 3879 control	RR: 0.51 (95% CI 0.34–0.76)
Salissou 2017	Niger	SP+AQ vs nothing	3-59 mo	4	241 SMC, 241 control	RR: 0.28 (95% CI 0.19-0.42)

SP: sulfadoxine-pyrimethamine; AQ: amodiaquine; SMC: seasonal malaria chemoprevention

Incidence of hospitalization for any cause in non-randomized studies

Two studies reported incidence of severe malaria among children < 5 years of age receiving three to four cycles of SP+AQ (Issiaka 2020, Salissou), both based on reports by guardians of children during cross sectional surveys. While Issiaka 2020 reports results comparable to results from randomized studies, Salissou 2017 reports an effect larger than that of randomized studies.

Table SF1.8. Incidence of all-cause hospitalization among children < 5 years of age, non-randomized studies

Study IDs	Country	Drug	Age group	# of cycles	N	Risk ratio
Issiaka 2020	Mali	SP+AQ vs nothing	3-59 mo	4	2759 SMC, 3879 control	RR: 0.58 (95% CI 0.41-0.81)
Salissou 2017	Niger	SP+AQ vs nothing	3-59 mo	4	241 SMC, 241 control	PR: 0.17 (95% CI 0.09-0.31)

SP: sulfadoxine-pyrimethamine; AQ: amodiaquine; SMC: seasonal malaria chemoprevention

All-cause mortality among children < 5 years of age in non-randomized studies

Three studies reported all-cause mortality among children < 5 years of age receiving 3-4 cycles of SMC in non-randomized designs, one that reported SP+AQ and DP (Zongo 2015), and two that reported SP+AQ Issiaka 2020, Salissou 2017). With the exception of the DP arm in Zongo 2015, the effect size was greater than that reported for randomized designs.

Table SF1.9. Incidence of all-cause mortality among children < 5 years of age, non-randomized studies

Study IDs	Country	Comparator	Age group	# of cycles	N	Risk ratio
Zongo 2015A	Burkina Faso	SP+AQ vs nothing	3-59 mo	3	742 SP+AQ, 250 control	RR: 0.67 (95% CI 0.06-7.43
Zongo 2015B	Burkina Faso	DP vs nothing	3-59 mo	3	757 DP, 250 control	RR: 1.32 (95% CI 0.5-11.82)
Issiaka 2020	Mali	SP+AQ vs nothing	3-59 mo	4	2759 SMC, 3879 control	RR 0.44 (95% CI 0.22–0.91)
Salissou 2017	Niger	SP+AQ vs nothing	3-59 mo	4	241 SMC, 241 control	RR: 0.54 (95% CI 0.22-1.33)

SP: sulfadoxine-pyrimethamine; AQ: amodiaquine; SMC: seasonal malaria chemoprevention; DP: dihydroartemisinin-piperaquine
